# Environmental contamination with polycyclic aromatic hydrocarbons and contribution from biomonitoring studies to the surveillance of global health

**DOI:** 10.1007/s11356-024-34727-3

**Published:** 2024-08-29

**Authors:** Joana Teixeira, Cristina Delerue-Matos, Simone Morais, Marta Oliveira

**Affiliations:** REQUIMTE/LAQV, ISEP, Polytechnique of Porto, Rua Dr. António Bernardino de Almeida 431, 4249-015 Porto, Portugal

**Keywords:** Environmental pollution, Polycyclic aromatic hydrocarbons, Biota, Biomonitors, Human exposure, One Health

## Abstract

This work presents an integrated overview of polycyclic aromatic hydrocarbons’ (PAHs) ubiquity comprising environmental contamination in the air, aquatic ecosystems, and soils; characterizes the contamination in biota; and identifies main biomonitors and human exposure to PAHs and associated health risks. Urban centers and industrial areas present increased concentrations in the air (1344.4–12,300 versus 0.03–0.60 ng/m^3^ in industrial/urban and rural zones) and soils (0.14–1.77 × 10^6^ versus 2.00–9.04 × 10^3^ versus 1.59–5.87 × 10^3^ ng/g in urban, forest, and rural soils), respectively. Increased concentrations were found in coastal zones and superficial waters as well as in sediments (7.00 × 10^4^–1.00 × 10^9^ ng/g). Benzo(a)pyrene, a carcinogenic PAH, was found in all environmental media. Mosses, lichens, tree leaves, bivalves, cephalopods, terrestrials’ snails, and honeybees are good biomonitors of biota contamination. More studies are needed to improve characterization of PAHs’ levels, distribution, and bioaccumulation in the environmental media and assess the associated risks for biota and human health. Actions and strategies to mitigate and prevent the bioaccumulation of PAHs in the environment and trophic chains toward the WHO’s One-Health Perspective to promote the health of all ecosystems and human life are urgently needed.

**Supplementary Information:**

The online version contains supplementary material available at 10.1007/s11356-024-34727-3.

## Introduction

The chemical pollution crisis is an undeniable worldwide reality that severely impacts environmental and human health. Recently, the World Health Organization (WHO) formed the One Health High-Level Expert Panel (OHHLEP) that defined the One Health Perspective - a unifying approach to sustainably balance and optimize the health of people, animals, and ecosystems, and recognized the close link and inter-dependent relation between the health of all ecosystems and human health (WHO 2022).

Polycyclic aromatic hydrocarbons (PAHs) are a group of more than a hundred organic compounds with two or more fused aromatic rings arranged in different configurations that are widespread in the environment as complex chemical mixtures (Zhang et al. 2022a). These compounds are semi-volatile and highly lipophilic, being also resistant to heat, corrosion, and degradation (Zhang et al. 2022a). PAHs integrate the list of persistent organic pollutants (POPs) (United Nations Economic Commission for Europe 1998) and are released into the air, water resources, and soils via different natural (e.g., volcanic eruptions) but principally through several anthropogenic emission sources (Carex Canada 2021; Krzyszczak and Czech 2021; Rovira et al. 2021). The primary sources are the incomplete burning of fuel, crude and refined petroleum, coal, wood, and biomass (Dat and Chang 2017; Lawal and Fantke 2017; Patel et al. 2020). Over the last years, the number of occurrences and severity of wildfires have progressively increased all over the world in part as a consequence of global warming and climatic changes, principally during heat waves (Bowman et al. 2019; Dupuy et al. 2020; Halofsky et al. 2020; Miezïte et al. 2022; Turco et al. 2019). Despite the recognized socio-economic impact of large and megafires, there is also a great concern related to the environmental contamination caused by PAHs among other hazardous pollutants released during wildfires, and the associated risks and health effects on the environment and exposed populations (Akdis and Nadeau 2022; Barros et al. 2023c; D’Evelyn et al. 2022; Ghetu et al. 2022). PAHs are used as intermediates in several industrial processes, namely pharmaceutical industries, the production of plasticizers and thermosetting plastics, drying agents, photographic products, pigments, and lubricant materials (Jameson 2019). Some PAHs, *e.g.,* pyrene (Py) and anthracene (Ant), are widely used in the synthesis of chemicals that are intermediates in the production of dyes and their precursors (United Nations Environment Programme and WHO 2013). Therefore, anthropogenic sources of PAHs include industries (e.g., those dedicated to the extraction and exploitation of petroleum derivatives, coke and aluminum production, power plants, and smelters), waste incinerators, transportation, oil spills, residential heating, and the burning of petroleum-derived products (Abdel-Shafy and Mansour 2016; Berthiaume et al. 2021; Patel et al. 2020). Consequently, PAHs are important pollutants, principally at urban and industrialized areas (Oliveira et al. 2019).

Among the hundreds of PAHs emitted to the environment, 16 are included in the list of priority pollutants, namely naphthalene (Naph), acenaphthylene (Acen), acenaphthene (Ace), fluorene (Flu), phenanthrene (Phen), Ant, fluoranthene (Fln), Py, benz(a)anthracene (BaA), chrysene (Chry), benzo(b)fluoranthene (BbFln), benzo(k)fluoranthene (BkFln), benzo(a)pyrene (BaP), dibenz(a,h)anthracene (DBahA), benzo(g,h,i)perylene (BghiP), and indeno(1,2,3-c,d)pyrene (Ind) (USEPA 2005) (Online Resource 1). Among these compounds, BaP is classified by the International Agency for Research on Cancer (IARC) as a known carcinogenic to humans, being also recognized as a genotoxic, mutagenic, epigenetic, teratogenic, and neurotoxic substance (Bukowska et al. 2022). Naph, BaA, Chry, Ind, and the isomers BbFln, BjFln, and BkFln are classified as possible carcinogens while DBahA and dibenzo(a,l)pyrene (DBalPy) are considered probable carcinogens to humans (IARC 2002; IARC 2010a, b). Recently, IARC evaluate the carcinogenicity of Ant as being possibly carcinogenic to humans (group 2B) (Cattley et al. 2023). Despite not being included in the list of priority pollutants, there are other PAHs also classified as probable (e.g., cyclopenta(c,d)pyrene) and possible (e.g., benz(j)aceanthrylene, benzo(c)phenanthrene, dibenzo(a,i)pyrene, and 5-methylchrysene) carcinogens to humans (IARC 2002, 2010b).

The environmental contamination with PAHs is a serious concern and it has direct implications for the health of ecosystems as well as for human health. So far, different authors revised the occurrence, main sources, and distribution of PAHs in some environmental media; however, an integrated and broad overview of the environmental ubiquity of these compounds remains limited. Data related to environmental and human exposure to PAHs via (bio)monitoring studies remain dispersed. To the best knowledge of these authors, there is a need to reunite the available literature on PAH ubiquity in the air, water resources, and soil and revise the information on environmental and human biomonitoring studies (HBM) to assess the adverse effects of PAHs on the environment, biota, and potential health risks for human health. To reply, this aims of this work are i) to present a global description of environmental contamination with PAHs in the air, aquatic ecosystems, and soil and identify the main sources of pollution; ii) to identify PAHs and describe its levels in biota through environmental biomonitoring studies; iii) to identify main PAH metabolites in human biological fluids and characterize available human surveillance programs; and iv) to make an overview of the health risks associated with human exposure to PAHs. This work converges the much-needed data on environmental contamination and human exposure to PAHs, which will identify additional research needs. It is believed that compiled data will raise awareness and support stakeholders and international agencies in the implementation of preventive and mitigation strategies, thus contributing to promote the envisaged WHO One-Health Perspective.

## Materials and methods

Information related to the presence of PAHs in different environmental matrices (air, aquatic systems, soil, and biota) and HBM studies assessing exposure to PAHs was searched in the literature. The search was done in different scientific databases (Scopus, PubMed, Science Direct, and Scielo) with the combination of at least two of the following keywords: PAHs, environmental pollution, air, aquatic systems, soil, environmental biomonitoring, human biomonitoring; the Boolean symbol “AND” was always used between keywords to make the search broader and more complete. Due to the high number of scientific papers available in the literature, the authors decided to consider the data published in the last 6 years (January 2017–December 2022). The following inclusion criteria were taken into consideration: i) to be a review work written in English; ii) to report the contamination with PAHs in environmental media; iii) to characterize exposure to PAHs in biota and humans. The abstract of all the reviews collected was screened after the elimination of duplicates. Data extraction was only performed from published tables and text; information presented in figures was not retrieved. The studies focused on food safety with PAH contamination and/or human exposure to PAHs via ingestion of foods were not considered. In the absence of reviews focusing on specific subjects, *e.g.*, biomonitoring assays performed in living organisms including humans, the most recent and relevant original articles were considered. Overall, data was retrieved from 59 reviews and 124 research papers.

## Environmental contamination

**Fig. 1:**
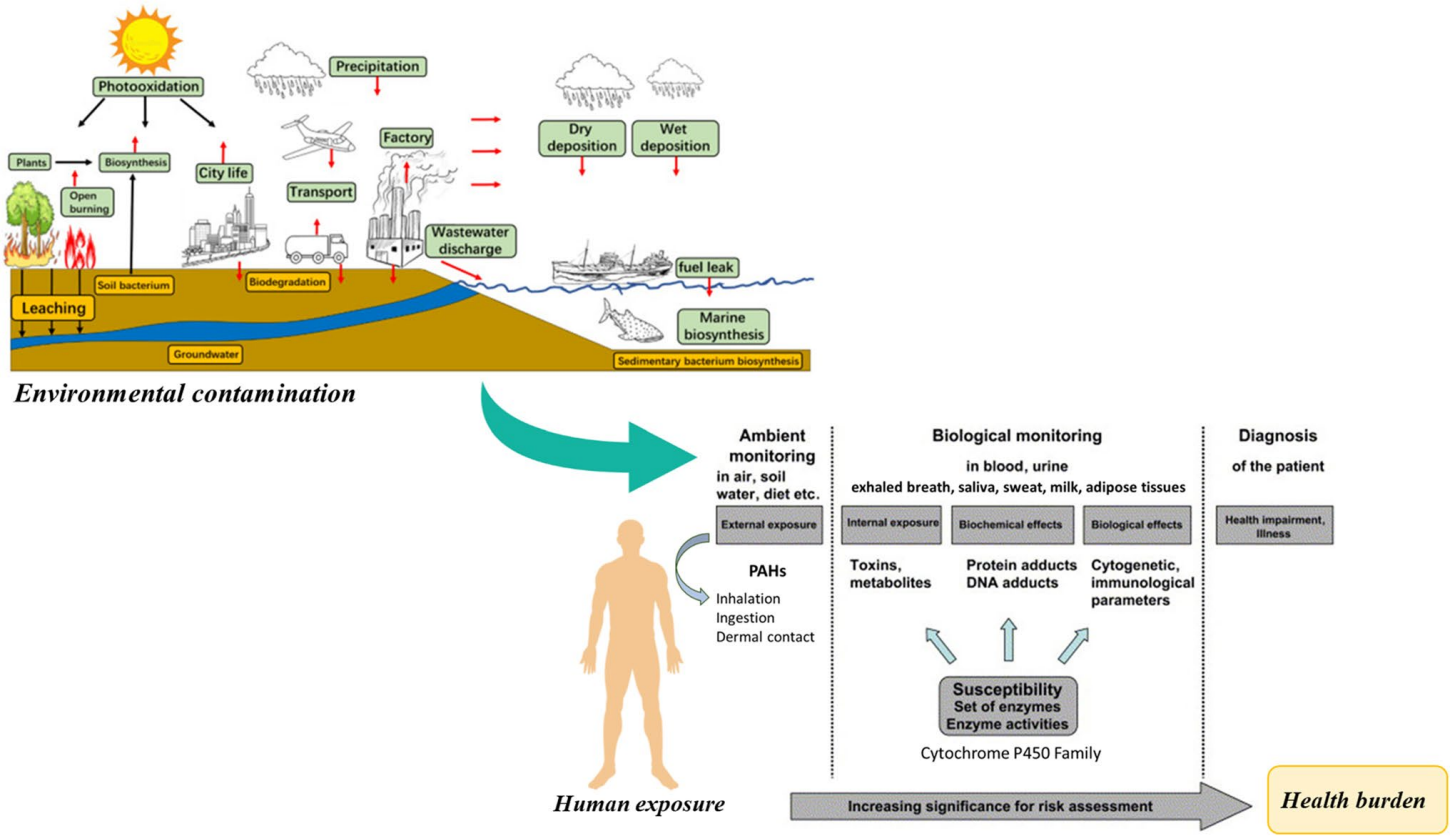
Sources of ambient (air, water resources, and soil) PAHs, transport processes in the environment, and contribution of environmental contamination to human exposure and health burden (adapted from Sun et al. (2021) and Angerer et al. (2007), copyright (2023), with permission from Elsevier)

Environmental pollution and climate change are within the biggest concerns among the worldwide population (European Comission 2017). The main route for environmental distribution and transboundary deposition of PAHs is the air (Abdel-Shafy and Mansour 2016). Airborne gaseous/particulate PAHs can be transported over long distances and deposited through dry or wet processes, including precipitation, or dissolution associated with wet accumulation of particles on aquatic resources and soils (Srogi 2007; Campos et al. 2019; Campos and Abrantes 2021; Srogi 2007). PAHs can also reach superficial waters by urban run-off containing compounds deposited on the surface of buildings, roads, and vegetation, municipal and industrial effluents, and by spillage or leakage of petroleum-derived products (Srogi 2007). Additionally, superficial water pollutants can infiltrate through the soil and reach underground waters that could also end up on seawater. Precipitation by dry or wet deposition, oil-derivatives spills, and fires are the main pathways for the input of PAHs into the soil surface (Hussain et al. 2018). The predominant mechanisms for the dispersion of PAHs in soils are volatilization, irreversible sorption, leaching, assimilation by plants, and biodegradation (Hussain et al. 2018). PAHs present in the soils and sediments are transported to surface and groundwaters, being later assimilated by different species of animals and plants (Abdel-Shafy and Mansour 2016; Patel et al. 2020). Distribution and consequent bioaccumulation of PAHs, in the environment and organisms, are strongly dependent on their physicochemical properties, namely molecular weight, non-polarity, and hydrophobicity, and on the type of interactions established with the surrounding media (Krzyszczak and Czech 2021). PAHs with two to three aromatic rings have low molecular weights and tend to be more volatile and soluble in water, while compounds with five and more benzene rings present reduced water solubility and volatility. The physical state and partition of PAHs are strongly dependent on ambient temperature (Krzyszczak and Czech 2021). Moreover, the transformation of PAHs in the environment originates derivatives containing nitrogen, oxygen, and sulfur in the aromatic rings (Krzyszczak and Czech 2021). The degradation of PAHs is slow and promotes the (bio)accumulation in the environment and on living organisms (Hussain et al. 2018; Krzyszczak and Czech 2021). Thus, PAHs are widely distributed through different environmental matrices, and they are found as a complex mixture of chemical compounds simultaneously in the air, aquatic resources, soils, and biota (Fig. 1).

### Air

Atmospheric pollution, including particulate matter (PM), nitrogen and sulfur oxides, ozone, volatile organic compounds including PAHs, among others, is among the most important environmental risks to all ecosystems and to human health, principally in rapidly developing and industrializing low- and middle-income countries (EEA 2020; Landrigan et al. 2018). IARC classified air pollution as a whole and PM as carcinogens to humans (IARC 2013). Airborne PAHs are found in the gas phase and can be adsorbed/absorbed on PM, whereby their distribution within the gas and particulate phases is dependent on the volatility of the compound, ambient temperature, and relative humidity (Oliveira et al. 2019; Wallace et al. 2020) (Online Resource 1). Higher concentrations of PAHs in both gas and particulate phases have been reported near large urban centers with high population density, mostly in areas located near industrial complexes and/or busy roads (EEA 2020; Kumar et al. 2020; Ofori et al. 2020; Oliveira et al. 2019; Rovira et al. 2021; Srogi 2007; Wallace et al. 2020; Zhang et al. 2022a). Evidence is progressively emerging from regions all over the world, highlighting the strong contribution of urbanization to human exposure to PAHs (Alvi et al. 2018; Du and Jing 2018; Manzetti 2013). Concerning seasonal variations, concentrations of atmospheric PAHs are predominantly higher in the cold season due to the use of home heating processes, higher dependency on diesel/gasoline vehicles, increased atmospheric stability, and reduced photolysis effects (Liu et al. 2022). The range of total PAHs reported in the ambient air retrieved from the selected reviews is presented in Online Resource 2. Overall, the lowest values of total PAHs were found in the air of rural areas (0.03–0.60 ng/m^3^), while the highest concentrations were reported in the air of industrial and intensive urbanized regions (1344.4–12,300 ng/m^3^). The BaP was found in the ambient PM at levels ranging between 0.01 and 15.0 ng/m^3^, while other possible/probable carcinogenic PAHs varied between 0.01 and 231 ng/m^3^ for Naph, 0.01 and 105 ng/m^3^ for Chry, and from 6.0 × 10^−3^ to 57.0 ng/m^3^ for DBahA (Online Resource 3). The last report released by the European Environmental Agency (EEA) revealed that 15% of the European population living in urban areas were exposed to annual BaP air levels above the available guideline (1 ng/m^3^) (EEA 2020). About 75% of that population was exposed to annual air values above the reference level of 0.12 ng/m^3^ defined by WHO (EEA 2020). Gaseous and PM-bound PAHs are transported across long distances away from the original source; however, the gaseous compounds are expected to be less stable (< 24 h) (WHO 2021). PM-bound PAHs, including possible/probable carcinogenic compounds, contribute to the carcinogenicity and mutagenicity of atmospheric PM, specifically if PM-bound BaP levels exceed the limit of 1.0 ng/m^3^ which can induce DNA damage (WHO 2010).

In the last years, political, media, and public interests in air quality issues have been growing, reflecting citizen science initiatives engaged in supporting air quality monitoring and actions targeting public awareness and behavioral changes (EEA 2020). These measures have allowed growing support and demand for actions to improve air quality.

Despite contributing to the health risks associated with air pollution, PAHs also contribute to damage materials, artworks, and cultural buildings through chemical reactions that promote corrosion (e.g., acidifying compounds), biodegradation, soiling (e.g., particles), and weathering/fading of colors (e.g., ozone) (EEA 2020; Slezakova et al. 2013). Once in the atmosphere, PAHs can undergo photochemical and complex physicochemical reactions with other pollutants (e.g., ozone, sulfur dioxide, and nitrogen oxides) that will originate nitrated, oxygenated, sulfated, and hydroxylated PAH derivatives, which are yet poorly characterized (Kameda 2011; Krzyszczak and Czech 2021). Airborne PAHs and derivatives can suffer degradation (e.g., the rapid photooxidation of gaseous compounds) and/or undergo dry and wet deposition into natural ecosystems (vegetation, water resources, and soils) and bioaccumulate in plants and other organisms (Fig. 1) (Haritash and Kaushik 2009; Kim et al. 2013; Srogi 2007).

### Aquatic ecosystems

**Fig. 2:**
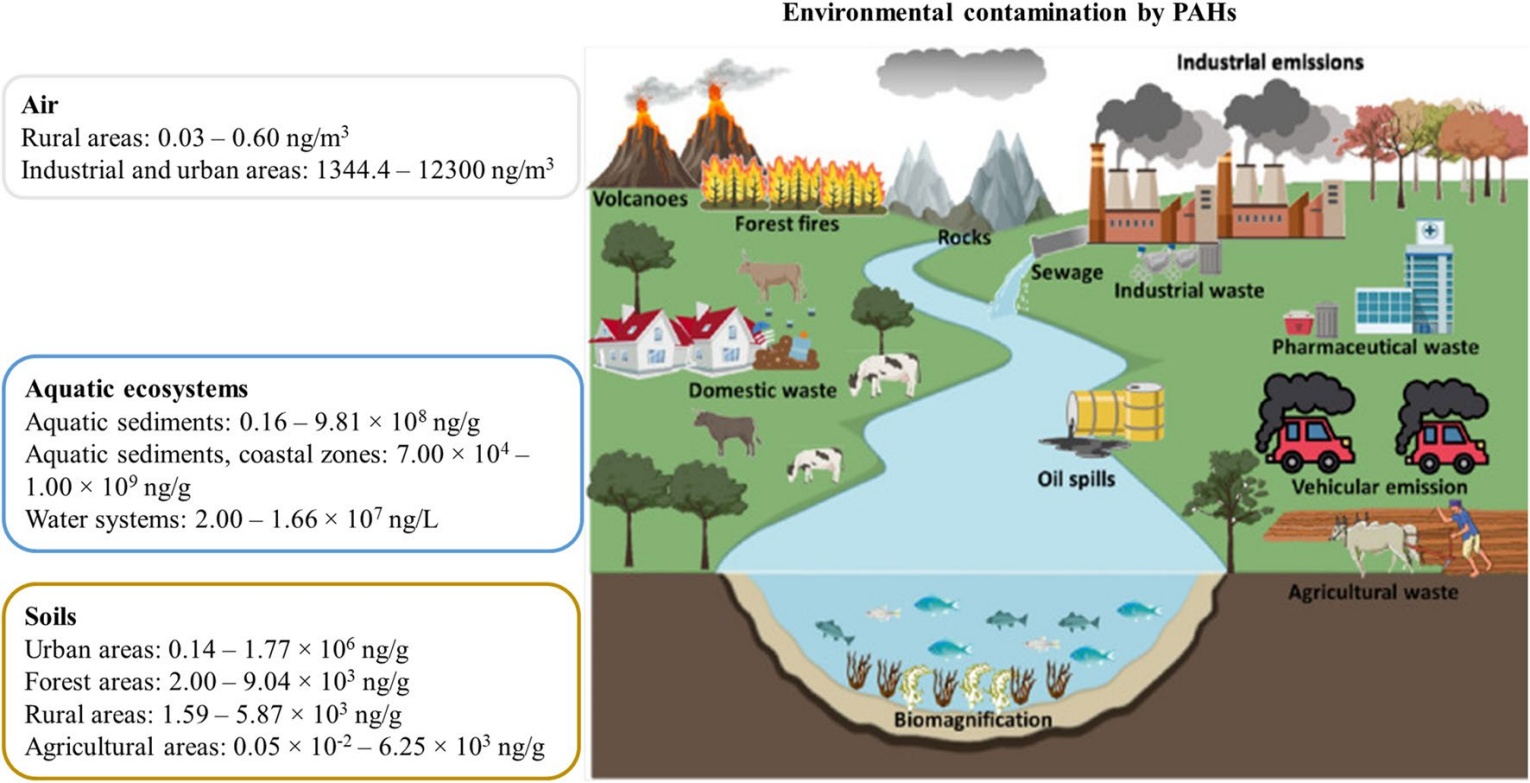
Environmental ubiquity of PAHs: overall concentrations in the air, water resources, and soils (adapted from Vandana et al. (2022), copyright (2023), with permission from Elsevier)

Water pollution, the second most concern type of ambient pollution, comprises unsafe water sources and/or inadequate sanitation (Landrigan et al. 2018). Overall, waterborne PAHs can be originated from petroleum-derived products/materials (petrogenic), incomplete combustion processes (pyrogenic) during anthropogenic activities, natural metabolism of some aquatic organisms (biogenic), and processes that cause the transformation of sediments (diagenetic) (Honda and Suzuki 2020; Maletic et al. 2019). The contamination of water resources is intensified in regions near large industrial complexes (e.g., chemical/petrochemical) mostly because discarded solid and aqueous wastes are not efficiently treated, and they could reach superficial and underground water resources (Ambade et al. 2021a, 2021b; Landrigan et al. 2018). Literature reports levels of total PAHs ranging between 0.16 and 9.81 × 10^8^ ng/g in aquatic sediments, and 2.00 and 1.66 × 10^7^ ng/L in water systems (Fig. 2; detailed information is presented in Online Resource 4). Jesus et al. (2022) reported the lowest levels of total PAHs being these values found in a river basin (2.00 to 1.49 × 10^4^ ng/L). Concentrations of PAHs are predominantly higher in aquatic sediments from coastal zones where urban and industrial activities are predominant (7.00 × 10^4^ to 1.00 × 10^9^ ng/g) (Du and Jing 2018; Ghandourah 2022; Maletic et al. 2019; Ofori et al. 2020). PAHs are known for their low affinity to water and their solubility decreases with the increase in molecular weight (Online Resource 1). Therefore, the lighter PAHs are mostly found dissolved in the water column while heavier compounds have a high capacity to adsorb on suspended particles as well as on non-polar matrices, which promotes their bioaccumulation in phytoplankton and aquatic sediments (Du and Jing 2018; Maletic et al. 2019; Thuy et al. 2018). PAH exchange and distribution between air–water-sediment explains their long-range contamination within water resources (Du and Jing 2018). Underground waters tend to have lower levels of PAHs than superficial waters because groundwater is naturally filtered as it crosses through various soil matrices, mainly in the layers richer in organic matter, being also less affected by anthropogenic pollutants (Srogi 2007). Evidence reports the presence of significantly higher concentrations of PAHs in phytoplankton, the primary resource and the most spatially extensive on food chains in nature, comparatively with the levels reported in seawater (Thuy et al. 2018). Bioaccumulation of PAHs in phytoplankton is propagated and magnified throughout the food chain and reach all trophic levels, including human (Thuy et al. 2018). Moreover, waterborne PAHs enter in trophic chains and tend to bioaccumulate in the fatty tissues of fishes and mollusks, principally in bivalves, cephalopods, and crustaceans among other benthos invertebrates (Du and Jing 2018; Honda and Suzuki 2020; Ofori et al. 2020; Oliveira et al. 2018; Thuy et al. 2018; Torrinha et al. 2014). Low molecular weight PAHs have more significant acute toxicity to aquatic organisms than high molecular weight compounds (Duan et al. 2015; Honda and Suzuki 2020; Thuy et al. 2018). Sediments in waterbeds (lakes, rivers, and seas/oceans) are the ultimate water sink that reflects the history of deposited pollutants, and they can be remobilized upward and re-suspended in the water column (Du and Jing 2018). Concentrations of PAHs in aquatic sediments tend to be higher than in the water column due to the low solubility and high hydrophobicity of compounds (Jesus et al. 2022; Robin and Marchand 2022). Once at the system water–sediment and depending on local physicochemical properties, PAHs are associated with particles rich in organic matter among other materials present in sediments (Jesus et al. 2022; Maletic et al. 2019; Robin and Marchand 2022). Therefore, sediments from high-polluted waters are a permanent source of pollution to benthos invertebrates, which will accumulate PAHs and other toxic pollutants, mainly in lipid-rich tissues (Maletic et al. 2019; Mearns et al. 2019; Jesus et al. 2022; Thuy et al. 2018). Regarding the reported levels of possible and probable carcinogenic PAHs, the literature reveals that BaP has been found in both superficial waters (1.0 × 10^−2^–4.30 × 10^5^ ng/L) and aquatic sediments (0.02–64,000 ng/g) (Online Resource 3). Other PAHs, e.g., Naph (1.0 × 10^−2^–1.00 × 10^6^ ng/L; 0.05–781 ng/g), BaA (2.0 × 10^−2^–4.30 × 10^5^ ng/L; 0.03–43,000 ng/g), benzofluoranthene isomers (1.0 × 10^−2^–7.40 × 10^5^ ng/L; 0.04–54,000 ng/g), DBahA (1.0 × 10^−2^–2.00 × 10^5^ ng/L; 0.01–9000 ng/g), and Ind (2.0 × 10^−2^–2.00 × 10^5^ ng/L; 0.09–57,000 ng/g) have also been reported in superficial waters and aquatic sediments, respectively (Online Resource 3).

### Soils

Soil pollution is of particular concern in urban areas mostly due to diffuse contamination, a continuous and long-term source of pollution. Kariyawasam et al. (2021) reported that 90% of environmental PAH deposition is in soils and sediments. Soils act as a storage of environmental pollutants since they retain contaminants for long periods of time (Cachada et al. 2016). The accumulation of pollutants in soils changes their physicochemical properties which will affect their productivity and quality, and it will act as a source of contamination for local animals and plants, agricultural fields, and surrounding water resources (Cachada et al. 2016). A high-contaminated soil can contribute to toxic effects on terrestrial invertebrates (Abdel-Shafy and Mansour 2016). The PAHs present in contaminated soils can be assimilated by the roots of plants, be distributed, and accumulated on the leaves, fruits, and seeds of a countless number of species which are the main food supply of several organisms, including insects, birds, terrestrial snails among other invertebrates, rabbits, hares, moles, and various mammals (e.g., cow, sheep, horse, and goat). As the food chain evolves, the PAHs assimilated from the soil will reach higher trophic levels including the human diet. Therefore, the dietary uptake of soils contaminated with PAHs can represent an important route of exposure for plants, animals, and human diet (Jesus et al. 2022). The potential risks to the environment and human health caused by soil contamination are dependent on the heterogeneity and complexity of the matrix, the presence of multiple diffuse sources, and mixtures of pollutants that can act synergistically (Cachada et al. 2016).

The deposition of PAHs in sediments occurs through the accumulation of PM-bound PAHs in water reservoirs (Kariyawasam et al. 2021). Organic matter and clay content of soil influence the distribution of PAHs as well as their solubility, vapor pressure, lipophilicity, octanol–water partitioning coefficient, and distribution coefficient across sediments (Hussain et al. 2018; Wallace et al. 2020) (Online Resource 1). Factors such as hydrophobicity, stability of the chemical structure, and association with organic matter contribute to a higher persistence of PAHs in soils (Hussain et al. 2018). Soils rich in organic matter present increased sorption of PAHs (Maletic et al. 2019). Levels of total PAHs reported in urban soils varied between 0.14 and 1.77 × 10^6^ ng/g (Fig. 2; detailed information is presented in Online Resource 5). Other authors compiled the contamination of PAHs in the forest (2.00 to 9.04 × 10^3^ ng/g), rural (1.59 to 5.87 × 10^3^ ng/g), and agricultural (5.00 × 10^−2^ to 6.25 × 10^3^ ng/g) soils (Online Resource 5). Moreover, different authors demonstrated the increased concentrations of PAHs in superficial layers of urban and forest soils comparatively with rural and agricultural lands (Cachada et al. 2016; Kariyawasam et al. 2021; Krzyszczak and Czech 2021; Liu et al. 2022; Zhang et al. 2022a). Overall, levels of BaP ranged from 4.0 × 10^−3^ to 0.34 × 10^3^ ng/g, reaching maximum values in agricultural soils (Online Resource 6). Other possible/probable PAHs were also found in soils: Naph (up to 0.97 × 10^3^ ng/g), BaA (up to 0.36 × 10^3^ ng/g), benzofluoranthene isomers (up to 0.61 × 10^3^ ng/g), Chry (up to 0.62 × 10^3^ ng/g), DBahA (up to 0.11 × 10^3^ ng/g), and Ind (up to 0.31 × 10^3^ ng/g) (Online Resource 6). The presence of PAHs in urban soils is globally associated with anthropogenic sources (e.g., domestic heating, industrial activity, and traffic emissions) (Dai et al. 2022; Liu et al. 2022). PAHs with low molecular weights present higher solubility and volatility, thus being naturally removed by physicochemical and biological processes (Maletic et al. 2019). Heavier compounds tend to accumulate in soils and present increased bioavailability for living organisms (Idowu et al. 2019). For example, the PM-bound PAHs present on the ashes of wildfires affect the quality of burned soils, its fertility, and productivity since several modifications occur in the chemical properties (e.g., pH, organic matter, humic acid content, and hydrophobicity) (Agus et al. 2019; Campos and Abrantes 2021; Certini et al. 2021). Transport of PAHs from burned soils to water resources occurs by surface runoff, thus leading to the introduction of ash and soil particles into aquatic ecosystems, affecting the surrounding aquatic food chain and water resources.

Lands from plateau and tropical climates usually present a lower concentration of PAHs due to a higher incidence of compounds with low molecular weight since there is an increased incidence of low-temperature combustion processes and biological sources, and reduced industrial activity (Krzyszczak and Czech 2021). Bioremediation processes present more promissory results in the treatment of areas contaminated with low molecular weight PAHs (e.g., Naph, Flu, Phen) while the treatment of places contaminated with complex mixtures, including heavier compounds (e.g., Chry, BaP, DBahA), needs more challenging approaches (Cachada et al. 2016; Daâssi and Qabil Almaghribi 2022; Duan et al. 2015; Kösesakal 2022; Salari et al. 2022). Catabolic processes can also represent effective approaches for the bioremediation of PAHs (Salari et al. 2022), being also under study the introduction of new technologies such as the use of biochar, micro-nano bubbles, reversible surfactants, and oxidation processes to reduce soil contamination with PAHs (Dai et al. 2022; Patel et al. 2022). Moreover, additional tools and technologies are needed to mitigate the contamination and dissemination of PAHs throughout the different ecosystems.

### Biota

The contamination of air, water resources, and soils acts as a source of exposure to different plants and animal species, including humans (Abdel-Shafy and Mansour 2016; Paris et al. 2018; Jesus et al. 2022). Environmental biomonitoring is based on the analysis of natural ecosystems and/or organisms to collect information, qualitative and quantitative, related to the presence of pollutants in a particular environment (Al-Alam et al. 2019). Biomonitors are living organisms naturally present in the environment that can accumulate PAHs in their tissues, thus reflecting the degree of contamination in their habitat (Al-Alam et al. 2019). The selection of biomonitoring species from biota is based on specificity, location, tissue type, metabolic capacity and accumulation ratio, occurrence, species richness, climatic and seasonal conditions, and biodiversity (Alshaarawy et al. 2013; Manzetti 2013; Semedo et al. 2014; Wallace et al. 2020). The environmental biomonitoring of PAHs has been applied in different ecosystems, being recognized as an economic technique with simple and easy implementation procedures (Holt and Miller 2010; NOAA 2022).

#### Plants

**Fig. 3:**
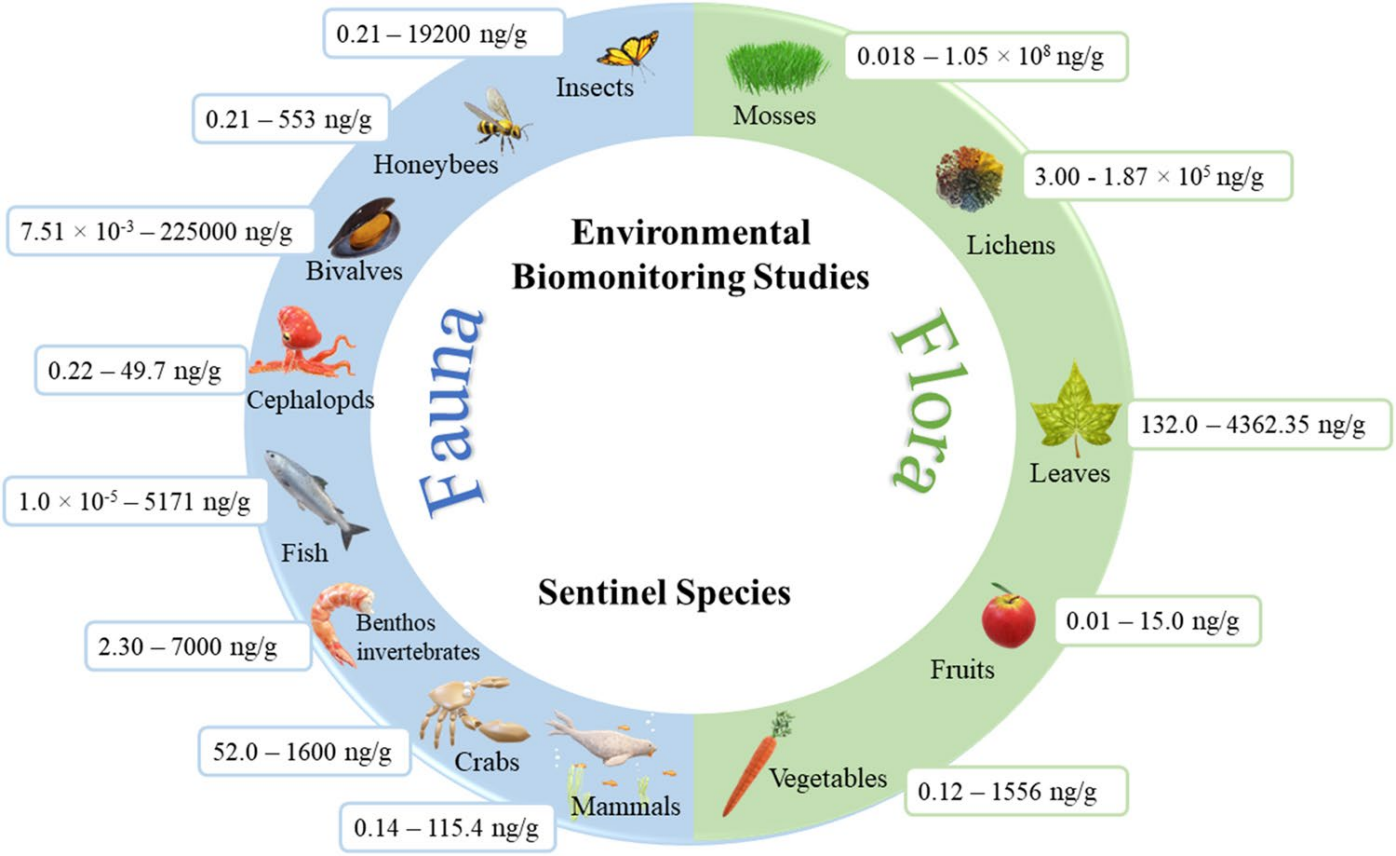
Environmental biomonitoring studies: overall concentrations of PAHs in biomonitors

The terrestrial plants can absorb PAHs from contaminated air, soils, and water resources, being further distributed through the different parts of plants (Abdel-Shafy and Mansour 2016). During the last decade, several studies addressed the biomonitoring of mosses, lichens, tree leaves, conifer needles, vegetables, and fruits (Abas 2021; Al-Alam et al. 2019; Bansal and Kim 2015; Huang et al. 2018; Kargar et al. 2017; Mukhopadhyay et al. 2020; Narayana Suvarapu and Baek 2017; Paris et al. 2018; Van der Wat and Forbes 2015; Wright et al. 2018). Mosses have been applied for the monitoring of atmospheric pollution in forests, mountains, rural/urban/industrial areas, and inaccessible sites (e.g., caves) (Al-Alam et al. 2019; Dolegowska et al. 2021; Mukhopadhyay et al. 2020; Narayana Suvarapu and Baek 2017). Overall, values ranged between 0.018 and 1.05 × 10^8^ ng/g in mosses collected in Hungary during the autumn season (Mukhopadhyay et al. 2020) (Fig. 3; details in Online Resource 7). Different possible/probable carcinogenic PAHs were found in mosses, including BaP (6.00–215 ng/g), Naph (41.0–105 ng/g), benzofluoranthene isomers (1.30–290 ng/g), Chry (5.00–313 ng/g), DBahA (0.40–49.0 ng/g), and Ind (0.70–208 ng/g) (Table 1). It has been demonstrated that mosses accumulate high molecular weight PAHs at their leaf surface (Al-Alam et al. 2019; Dolegowska et al. 2021; Narayana Suvarapu and Baek 2017). Some authors evaluated the potential of lichens to act as environmental bioindicators of forests, mountain regions, and areas with different levels of pollution (Abas 2021; Al-Alam et al. 2019; Dolegowska et al. 2021; Mukhopadhyay et al. 2020; Narayana Suvarapu and Baek 2017; Van der Wat and Forbes 2015). The range of total PAH levels found in lichens ranged between 3.00 and 1.87 × 10^5^ ng/g (Fig. 3 and Online Resource 7). As already observed in other sensitive species, different possible/probable carcinogenic PAHs were identified in lichens [BaP (2.11–1900 ng/g), Naph (2.00–5659 ng/g), BaA (3.38–344 ng/g), and DBahA (0.450–3060 ng/g) Table 1]. Among the different species of lichens that have been considered, fruticose lichens presented a higher ability to retain low molecular weight PAHs while foliose lichens were more prompt to absorb high molecular weight compounds (Van der Wat and Forbes 2015). An advantage of using mosses and lichens as environmental bioindicators is their ability to monitor areas with difficult access to humans (Huang et al. 2018; Wright et al. 2018). However, these authors are aware that other environmental factors including temperature, relative humidity, and contamination with other relevant pollutants can also affect these very sensitive species. Tree leaves can also be used as bioindicators, especially for air quality monitoring since they provide data concerning air-dispersed pollutants in ecosystems and tend to accumulate pollutants, including PAHs (Mandal et al. 2018). Overall, the concentrations of total PAHs in leaves varied between 132.0 and 4362.35 ng/g (Fig. 3). Kargar et al. (2017) characterized the concentrations of total PAHs in pine leaf leaves near an industrial area, reporting values ranging between 138.6 and 853.7 ng/g (Fig. 3 and Online Resource 7). Additionally, the levels of possible and probable carcinogenic PAHs observed in conifer needles and leaves ranged between 0.03–703 ng/g and 0.30 to 216 ng/g for BaP, 0.78 to 320 ng/g and 13.0 to 177 ng/g for Naph, and 0.04 to 287 ng/g and 1.50 to 537 ng/g for Chry, respectively (Table 1). Al-Alam et al. (2019) highlighted the use of different species (conifer needles, lichens, mosses) in environmental biomonitoring assays and presented conifer needles as the most promising choice for POP monitoring, especially PAHs. Those authors also referred that the simultaneous use of different species can be promising to obtain a more complete and wider characterization of the selected environment (Al-Alam et al. 2019). Vegetables (e.g., carrot, potato, spinach, and turnip) and some fruits (e.g., banana, tomato, apple, and grape) have been used in environmental biomonitoring studies as a tool to monitor PAHs (Bansal and Kim 2015; Paris et al. 2018). Overall, the reported concentrations of total PAHs in fruits and vegetables varied between 0.01 and 15.0 ng/g and from 0.12 to 1556 ng/g, respectively (Fig. 3). The apple (0.02–15.0 ng/g), grapefruit (12.01–12.34 ng/g), and kiwi (7.17–11.19 ng/g) were the more contaminated fruits, while apricot (0.01–0.40 ng/g), grape (0.34–0.79 ng/g), and gooseberry (0.01–0.03 ng/g) presented the lowest levels (Online Resource 8). Regarding vegetables, cabbage (0.03–438 ng/g), cucumber (0.04–1556 ng/g), and spinach (0.61–1139 ng/g) presented the highest levels of total PAHs, whereas endive (0.29–0.68 ng/g) and leek (0.12–0.79 ng/g) presented the lowest ones (Online Resource 9). Levels of BaP varied between 0.01 and 0.87 ng/g in fruits and from 0.01 to 9.80 ng/g in vegetables. Concentrations of possible/probable carcinogenic PAHs were also found in both fruits and vegetables: 0.01–4.05 ng/g and 0.01–590 ng/g for Naph, 0.01–1.78 ng/g and 0.01–35.0 ng/g for BaA, 0.01–2.71 ng/g and 0.03–45.0 for Chry, and 0.01–2.88 ng/g and 0.01–25.0 ng/g for DBahA, respectively (Tables 2 and 3). Fruits and vegetables are contaminated through atmospheric pollution (predominant route), soil uptake, and autogenous biosynthesis. It was reported that tubers, stem-based plants, and leafy vegetables are very sensitive to environmental contamination with PAHs, principally those that are produced at the most polluted sites. Compared with fruits, vegetables tend to present higher concentrations of PAHs due to their direct uptake of these compounds from contaminated soils (Bansal and Kim 2015). Concerning fruits, evidence suggests that PAHs tend to accumulate more in ripe fruits, and in the peel compared to cores and pulp (Paris et al. 2018).

**Table 1:** Levels of possible/probable carcinogenic PAHs (expressed as range; ng/g) reported in mosses, lichens, conifer needles, and leaves [1, Huang et al. 2018; 2, Wright et al. 2018; 3, Abas 2021; 4, Dolegowska et al. 2021; 5, Al Alam et al. 2019]

PAH	Mosses		Lichens			Conifer needles				Leaves		
	[1]	[2]	[2]	[3]	[4]	[1]	[2]	[4]	[5]	[1]	[2]	[5]
Benz(a)anthracene	3.00–188	18.0–26.0	17.0–344	3.38–280	4.66–14.7	2.37–37.0	< 8.00–32.7	0.02–12.5	0.04–156	54.2–56.1	1.40–346	2.28–15.7
Benz(a)anthracene + Chrysene	nr	nr	11.5–132	1.20–133	nr	nr	nr	nr	nr	nr	nr	nr
Benzo(a)pyrene	6.00–215	< 12.0–31.0	< 12.0–64.0	2.11–1900	< 4.20–14.6	1.10–43.0	< 12.0	0.03–4.47	0.25–703	1.00–216	0.30–216	1.17–14.4
Benzo(b)fluoranthene	6.00–290	64.0–79.0	2.80–136	5.40–950	2.19 –19.9	2.95–54.0	< 3.00–12.3	0.04–8.29	0.53–5.08^a^	1.80–148	1.80–167	5.50–34.3^a^
Benzo(k)fluoranthene	1.30–110	31.0–36.0	0–103	2.10–1340	0.95–13.7	1.00–23.0	< 12.0	0.02–10.7	nd–79.0	0.20–37.0	0.20–98.8	1.86–16.6
Benzo(b + k)fluoranthene	nr	nr	nr	19–79	25.1–49.1	nr	nr	< 3.60	nr	nr	nr	nr
Benzo(b + j + k)fluoranthene	nr	nr	nr	31.1–192	nr	nr	nr	nr	nr	nr	3.03–127	nr
Chrysene	5.00–313	61.0–78.0	3.90–155	9.00–500	1.05–26.2	8.94–115	3.40–43.7	0.05–40.9	0.04–287	1.50–138	1.50–537	9.32–34.0
Chrysene + triphenylene	nr	nr	nr	64–159	nr	nr	nr	nr	nr	Nr	7.12–205	nr
Dibenz(a,h)anthracene	0.40–49.0	< 20.0	< 20.0–316	0.45–3060	< 4.90–1.91	1.00–6.00	< 20.0	< 4.90–40.9	0.05–18.8	0.10–60.9	0.10–60.9	0.62–26.9
Dibenz(a)anthracene	nr	nr	nr	11.3–80.9	nr	nr	nr	nr	nr	nr	nr	nr
Indeno(1,2,3-c,d)pyrene	0.70–208	39.0–46.0	0–83.0	1.73–75	< 3.40–225	1.20–36.0	< 20.0	< 3.40–36.6	nd–255	0.20–48.0	0.20–48.0	1.13–20.5
Indeno(1,2,3-c,d)pyrene + Benzo(g,h,i)perylene	nr	nr	203	8.90–263	nr	nr	nr	nr	nr	nr	nr	nr
Naphthalene	41.0–105	nr	7.70–5659	2.00–2860	nr	2.86–47.7	5.50–43.5	43.0–160	0.78–320	13.0–177	13.0–110	48.5–89.2

*nd*, not detected; *nr*, not reported

^a^ represents the sum of benzo(b)fluoranthene and benzo(j)fluoranthene

**Table 2:** Levels of possible/probable carcinogenic PAHs (expressed as range; ng/g) reported in different fruits (Paris et al. 2018)

PAH	Apple	Apricot	Banana	Cherry	Grape	Grapefruit	Gooseberry	Guava	Lemon	Mandarin
Benz(a)anthracene	< 0.01–0.20	< 0.01–0.56	< 0.01	< 0.01–0.76	0.03–0.25	1.33–1.78	nr	0.14–0.63	0.14–0.15	< 0.01–0.81
Benzo(a)pyrene	< 0.01–0.87	< 0.01–0.72	0.13–0.72	0.27–0.84	0.03–0.34	0.24–0.29	< 0.02–0.12	0.19	0.10–0.64	0.25–0.76
Benzo(b)fluoranthene	< 0.03–0.20	0.04–0.80	nr	nr	0.10–0.17	nr	nr	nr	nr	nr
Benzo(b + j)fluoranthene	nr	nr	nr	nr	nr	nr	< 0.03–0.32	nr	nr	nr
Benzo(k)fluoranthene	< 0.01–0.92	< 0.01–0.39	< 0.01	0.67–0.83	0.02–0.10	3.77–4.67	< 0.01–0.06	0.14–0.19	0.16–0.21	< 0.01–0.72
Chrysene	< 0.01–1.04	< 0.01–0.92	< 0.01	< 0.01–0.78	0.05–0.17	0.17–0.76	nr	0.23	0.13–0.16	< 0.01–0.52
Dibenz(a,h)anthracene	< 0.01–0.71	< 0.01–0.07	< 0.01–0.14	< 0.01–0.62	0.11	1.99–2.88	nr	nr	0.26–0.36	0.15–0.21
Indeno(1,2,3-c,d)pyrene	0.01–0.73	< 0.01–0.45	< 0.01–1.32	nr	0.04–0.07	0.14–0.69	< 0.21	nr	0.45–0.49	0.19–1.04
Naphthalene	< 0.01–4.05	< 0.01–0.62	< 0.01–0.44	< 0.01	0.19–0.37	< 0.01–0.81	< 0.52	0.17	0.43–0.89	0.39–0.46
PAH	Nectarin	Orange	Peach	Pear	Pineapple	Plum	Quince	Sapota	Strawberry	Kiwi
Benz(a)anthracene	< 0.01–0.71	< 0.01	< 0.01–0.46	0.20	0.01	nr	< 0.01–0.72	0.01	0.05–0.16	0.89–0.92
Benzo(a)pyrene	0.41–0.60	< 0.01	0.40–0.69	< 0.02–0.14	0.03	< 0.02–0.02	< 0.01	nr	0.03–0.15	0.72–0.87
Benzo(b)fluoranthene	nr	nr	nr	0.20	nr	nr	nr	nr	0.02–0.16	nr
Benzo(b + j)fluoranthene	nr	nr	nr	< 0.03	nr	< 0.03–0.03	nr	nr	nr	nr
Benzo(k)fluoranthene	0.37–0.42	< 0.01	0.15–0.47	< 0.01–0.10	0.09	nr	< 0.01–0.45	nr	0.01–0.07	0.56–0.92
Chrysene	< 0.01–0.71	< 0.01	< 0.01–0.36	nr	nr	nr	< 0.01–0.36	nr	0.29–0.87	1.32–2.71
Dibenz(a,h)anthracene	< 0.01–0.97	< 0.01	< 0.01–0.54	nr	nr	nr	-	nr	0.03	2.17–2.78
Indeno(1,2,3-c,d)pyrene	0.17–0.33	0.3	0.12–0.25	< 0.21	nr	< 0.21	< 0.01–0.69	nr	0.08	0.18–1.32

*nr*, not reported

**Table 3:** Levels of possible/probable carcinogenic PAHs (expressed as range; ng/g) reported in different vegetables (Paris et al. 2018)

PAH	Cabbage	Carrot	Cauliflower		Celery	Chili	Colocasia	Cucumber	Eggplant	Endive	Fenugreek	Garlic	Gourd
Benz(a)anthracene	0.04–5.46	< 0.01–5.40	nd–0.33		1.51–10.7	0.03	0.10–3.10	1.03–7.12	0.05–6.91	nr	0.02–0.66	0.31–0.92	0.24–0.84
Benzo(a)pyrene	0.02–5.69	0.01–3.64	nd–0.33		0.23–8.38	0.20	nr	0.01–9.80	0.02–5.22	0.01–0.03	0.05–0.43	0.34–0.72	0.01–2.11
Benzo(b)fluoranthene	0.01–0.92	0.02–2.69	nd–6.40		nr	nr	nr	0.01–1.12	0.78–9.32	0.05–0.10	nr	nr	0.87–1.33
Benzo(b + j)fluoranthene	nr	< 0.03–4.38	nr		nr	nr	nr	nr	nr	nr	nr	nr	nr
Benzo(k)fluoranthene	0.01–0.55	0.01–4.36	nd–0.17		0.21–0.48	nr	0.10	0.02–1.54	0.05–1.65	0.02–0.04	nr	0.11–1.36	0.57–0.71
Chrysene	0.03–15.0	0.04–4.75	nd–0.34		nr	nr	7.90	nr	0.13	0.19–0.39	0.12	nr	0.05–0.36
Dibenz(a,h)anthracene	0.02–15.0	< 0.01–1.98	nr		0.31–0.96	nr	nr	0.33–0.77	0.18–0.76	0.01	0.38	0.11–0.45	0.19–1.17
Indeno(1,2,3-c,d)pyrene	0.01–0.54	< 0.01–1.60	nd–0.20		0.23–0.39	nr	nr	0.47–0.82	nr	0.02–0.05	0.14	0.15–0.43	0.11
Naphthalene	0.03–90.0	0.01–5.50	0.60–2.69		0.48	1.17	2.90–11.7	0.70–264	10.1	1.62–3.78	1.36–3.65	0.32–0.46	1.11–10.5
PAH	Kale	Kohlrabi	Leek		Lettuce	Onion	Peas	Potato	Radish	Shallot	Spinach	Tomato	Turnip
Benz(a)anthracene	nd–0.70	0.19	0.38		0.14–35.0	nr	15.2	< 0.01–6.11	0.01–1.70	12.9	0.02–13.0	0.02–5.11	1.30–3.44
Benzo(a)pyrene	nd–0.31	0.04	nd–0.04		< 0.02–3.62	0.22–0.55	4.71	< 0.01–9.15	0.05	nr	0.12–2.45	0.01–7.57	0.08–2.10
Benzo(b)fluoranthene	nd–0.52	nd–0.15	nd–0.05		0.08–0.41	nr	14.1	0.02–1.67	0.01	11.6	0.30–0.89	0.02–9.40	0.04–2.34
Benzo(b + j)fluoranthene	nr	nr	nr		0.05–4.43	nr	nr	< 0.01–0.72	nr	nr	nr	nr	nr
Benzo(k)fluoranthene	0.16	nd–0.05	nd–0.07		< 0.01–3.53	0.18–0.85	4.73	< 0.01–4.04	0.02	11.1	0.10–0.87	0.01–0.46	0.02–0.73
Chrysene	nd–1.87	nd–1.07	nd–0.17		0.05–5.93	0.56–0.83	17.5	0.03–1.00	1.45–4.00	13.2	0.20–45.0	0.03–1.00	0.23
Dibenz(a,h)anthracene	0.05	0.04	0.01		0.02–25.0	0.34–0.93	nr	< 0.01–0.73	nr	nr	0.38–1.70	0.12–0.61	0.01–1.16
Indeno(1,2,3-c,d)pyrene	nd–0.28	nd–0.05	0.02		0.05–4.28	0.14–0.41	5.72	< 0.01–1.10	nr	nr	0.55–1.13	0.02–0.41	nr
Naphthalene	nr	nr	1.00–3.15		0.29–54.0	0.43–245	19.7	< 0.01–3.59	0.36–43.0	16.5	0.50–41.0	0.03–16.4	0.50
PAH	Scallion	Chinese cabbage		Cucumber	Cabbage	Tomato	Eggplant	Waxgourd	Celery	Potato	Carrot	Squash	Lettuce
Benz(a)anthracene	9.34	0.83	1.83–5.63		5.46	2.04–5.11	2.09–6.91	2.40–7.0	1.51–10.7	1.95–6.11	nr	nr	nr
Benzo(a)pyrene	7.20	0.98	4.94–9.80		5.69	4.05–7.57	2.80–5.22	2.89–7.45	5.19–8.38	< 0.02–9.15	< 0.02–0.41	< 0.02–0.05	< 0.02–2.97
Benzo(b + j)fluoranthene	nr	nr	nr		nr	nr	nr	nr	nr	0.05–0.72	nr	0.04–0.07	0.05–1.10
Benzo(k)fluoranthene	nr	nr	nr		nr	nr	nr	nr	nr	< 0.01–0.37	< 0.03–0.35	< 0.01–0.01	< 0.01–0.51
Chrysene	nr	nr	nr		nr	nr	nr	nr	nr	nr	0.01–0.66	nr	nr
Indeno(1,2,3-c,d)pyrene	nr	nr	nr		nr	nr	nr	nr	nr	< 0.21–1.60	nr	< 0.21	< 0.21

*nd*, not detected; *nr*, not reported

#### Animals

Some authors already highlighted that dietary uptake of soil is an important route of exposure to PAHs for some invertebrate species (Jesus et al. 2022). Further ingestion of these contaminated animals by predators from higher trophic levels will contribute to the dissemination of pollutants throughout the trophic chain. Environmental monitorization of PAHs with animals has emerged during the last years and available studies were performed with different species including benthos invertebrates, fish, amphibians, cephalopods, bivalves, terrestrials’ snails, birds, insects, and mammals (Fig. 3) (Al-Alam et al. 2019; Cunningham et al. 2022; Gomes et al. 2013; Sun et al. 2021; Thuy et al. 2018; Wallace et al. 2020).

Regarding aquatic ecosystems, the accumulation of PAHs in phytoplankton and microalgae has been demonstrated, highlighting their contribution to aquatic organisms’ bioaccumulation and biomagnification across different trophic levels (Thuy et al. 2018). Microalgae is an important bioindicator in coastal waters, although studies characterizing phytoplankton and microalgae as sentinel species remained limited (Thuy et al. 2018). Different authors already demonstrated the accumulation of PAHs in benthos invertebrates, amphibians, and fish (Gauthier et al. 2014; Honda and Suzuki 2020; Mearns et al. 2019; Pulster et al. 2020; Wallace et al. 2020). Overall, levels of total PAHs varied between 1.0 × 10^−5^ and 5171 ng/g for fish, 2.3 and 7000 ng/g for benthos invertebrates, 111 and 1050 ng/g for amphibians, and 7.51 × 10^−3^ and 225,000 ng/g for bivalves (Fig. 3; detailed information is presented in Online Resource 10). Levels of BaP in aquatic species have been reported at values ranging from 3.60 × 10^−4^ to 149 ng/g (Table 4). Moreover, the BaA (4.40 × 10^−4^–352 ng/g), Chry (1.00 × 10^−5^–348 ng/g), and Naph (1.00 × 10^−3^–104 ng/g) have also been described in aquatic species (Table 4). In these organisms, the uptake of low molecular weight PAHs occurs through direct contact with polluted water (e.g., via the gills, maternal transfer, and diet) while heavier compounds enter the organism via ingestion of sediments (e.g., burrowing, filter feeding, and ingestion). The seasonal distribution pattern of PAHs in muscle tissue of four fish species was evaluated by Recabarren-Villalón et al. (2021). Total PAH levels varied between 5.0 and 325.0 ng/g with predominance of petrogenic PAHs in the colder season (Recabarren-Villalón et al. 2021). Those authors demonstrated the use of fish species as useful sensitive bioindicators for the evaluation of environmental PAH contamination. Several bivalve species, including mussels, clams, and oysters, have also been extensively used as marine bioindicators of environmental contamination with PAHs (Thuy et al. 2018). Aguirre-Rubi et al. (2019) selected three different species of bivalves and evaluated the tissue content on PAHs, being Phen (0.49–173.0 ng/g), Fln (0.34–64.0 ng/g), Py (1–33.0 ng/g), benzo(b + k)fluoranthene (1.0–27.0 ng/g), and Chry (1.0–40.0 ng/g) the most predominant compounds. Those authors emphasized the importance of using bivalves as sentinel species to monitor the presence and distribution of PAHs as well as the ecosystem’s health.

**Table 4:** Levels of possible/probable carcinogenic PAHs (expressed as range; ng/g) reported in benthos invertebrates, fish, bivalves, and cephalopods [1, Honda and Suzuki 2020; 2, Srogi 2007; 3, Gomes et al. 2013; 4, Semedo et al. 2012; 5, Wright et al. 2018; 6, Power et al. 2021; 7, Waszak et al. 2021; 8, Quinete et al. 2020; 9, Provencher et al. 2020]

PAH	Benthos invertebrates	Fish		Bivalves	Cephalopds	
	[1]	[1]	[2]	[1]	[3]	[4]
Benz(a)anthracene	2.50–85.6	0.34	nr	4.40 × 10^−4^–352	< 0.07–0.70	0.04–2.85
Benz(a)anthracene + Chrysene	nr	nr	nr	0.96–109	nr	nr
Benzo(a)pyrene	6.40–89.8	nr	nr	3.60 × 10^−4^–149	< 0.09–0.22	0.11–2.32
Benzo(b)fluoranthene	19.0–138	nr	nr	2.50 × 10^−4^–255	nr	nr
Benzo(b + j)fluoranthene	nr	nr	nr	nr	< 0.30–0.70	0.52–4.71
Benzo(k)fluoranthene	5.70–48.1	nr	nr	8.20 × 10^−4^–120	< 0.04–0.13	0.04–3.19
Benzo(b + j + k)fluoranthene	nr	nr	nr	2.04–92.7	nr	nr
Chrysene	5.40–65.7	nr	1.00 × 10^−5^–2.00	5.80 × 10^−4^–348	< 0.05–4.62	0.20–24.9
Dibenz(a,h)anthracene	1.60–32.7	nr	nr	3.56 × 10^−3^–13.6	nr	0.78–2.95
Dibenzo(a,l)pyrene	nr	nr	nr	nr	< 0.20–0.53	nr
Indeno(1,2,3-c,d)pyrene	nr	nr	nr	2.00 × 10^−4^–51.3	< 0.06–1.01	0.18–4.65
Naphthalene	1.95–8.50	1.82—5.32	nr	1.00 × 10^−3^–21.7	< 0.70–3.53	0.36–104
PAH	Birds					Crab
	[5]	[6]	[7]	[8]	[9]	[5]
Benz(a)anthracene	nr	0.05–0.10	0.01–0.08	< 8.15	nd–72.4	30.0–23,667
Benzo(a)pyrene	0.07–0.10	5.00 × 10^−3^–0.03	0.10–0.20	< 8.15–20.3	nd–47.2	9873–22,921
Benzo(b)fluoranthene	nr	nr	0.04–0.40	< 16.3	nd–150	97.0–31,510
Benzo(k)fluoranthene	0.05–0.10	nr	0.20	< 8.15	nd–46.8	1057–31,039
Chrysene	nr	nr	0.02–0.10	< 16.3	nd–310	30.0–30,719
Dibenz(a,h)anthracene	0.08–0.10	0–0.01	0.03–0.30	< 8.15	nd–8.06	2347–133,678
Indeno(1,2,3-c,d)pyrene	nr	1.00 × 10^−3^–0.07	0.01–0.30	< 8.15–37.1	nd–34.5	3547–28,179
Naphthalene	< 0.33–1.10	nr	0.20–4.70	-	nd–529	9367–97,067

*nd*, not detected; *n.r.*, not reported

Cephalopods (e.g., octopus, squids, and cuttlefish) are predators that feed upon shrimps, crabs, and bivalve mollusks, among other organisms, and present a reduced capacity to metabolize PAHs, thus being very prone to bioaccumulation. Available evidence has demonstrated the potential of cephalopods to be used as marine sentinel species due to their high growth rates and short life expectancy (Semedo et al. 2012, 2014). Levels of total PAHs in cephalopods varied between 0.22 and 49.7 ng/g (Fig. 3 and Online Resource 10) being Ind (0.06–4.65 ng/g), Chry (0.05–24.9 ng/g), and Naph (0.36–104 ng/g) the predominant possible/probable carcinogenic PAHs (Table 4). Concentrations of PAHs and/or their metabolites are increased in the bile and fat tissues of lower trophic fish as well as on their edible tissues, thus promoting bioaccumulation into the marine food chain which might reach higher tropic levels including the human diet (Domingo and Nadal 2015; Gomes et al. 2013; Oliveira et al. 2020b; Yebra-Pimentel et al. 2015). Additionally, some authors also reported the presence of PAHs in phytoplankton (0.033 to 16,343 ng/g), and crabs (52.0 to 1600 ng/g) (Online Resource 10). Aquatic insects, birds (e.g., tree swallows, seagulls, cormorants), and some mammals (e.g., otters and seals among other species) can also present increased levels of PAHs due to their direct contact with polluted air and water in addition to the direct feeding from water diet (Wallace et al. 2020) (Table 4). For those species, the primary route of exposure to low molecular weight PAHs is direct contact with contaminated water, while the main exposure to high molecular weight PAHs is through ingestion of contaminated food (Wallace et al. 2020). This environmental contamination is more pronounced in coastal waters near industrialized sites and in areas directly affected by accidental oil spills (Abdel-Shafy and Mansour 2016; Adzigbli and Yuewen 2018; Hayakawa 2018; Pampanin 2017). The levels of total PAHs found in mammals (sea otters, harp seals, woodland caribou, moose, and grey wolf) varied between 0.14 and 115.4 ng/g (Fig. 3 and Online Resource 11). Birds are exposed to PAHs through the uptake of contaminated water, soil, and foods (e.g., seeds, leaves, fruits), although inhalation is mentioned as the main route of exposure (Power et al. 2021; Waszak et al. 2021). Biomonitoring studies including birds and mammals remain limited; however, evidence suggests increased levels of PAHs in species living near polluted sites (Al-Alam et al. 2019; Provencher et al. 2020; Power et al. 2021; Quinete et al. 2020; Waszak et al. 2021). Despite the fast metabolism of PAHs in the organism of birds, they tend to bioaccumulate PAHs in eggs (Power et al. 2021; Provencher et al. 2020; Wallace et al. 2020; Waszak et al. 2021; Wright et al. 2018). In this context, Power et al. (2021) reported a range level of total PAHs of 0.8–1.4 ng/g in seabird eggs. PAHs have been also detected in the liver, kidney, lung, brain, muscle, blood, and feces of different species of birds (Power et al. 2021; Provencher et al. 2020; Wallace et al. 2020; Waszak et al. 2021; Wright et al. 2018). Waszak et al. (2021) found the maximum concentrations in the lungs and kidneys (20 and 19 ng/g wet weight, respectively) of seabirds. Provencher et al. (2020) also reported total concentrations of PAHs (9.57–99.05 ng/g) in the liver of marine bird species.

Within the terrestrial ecosystem, different species have been included in environmental surveillance studies such as snails, insects including honeybees, birds (e.g., homing pigeons, buzzards, tawny owls), and mammals (e.g., woodland caribou, moose, gray wolf) (Al-Alam et al. 2019; Kargar et al. 2017; Mearns et al. 2019; Wallace et al. 2020; Wright et al. 2018). Some authors reported that terrestrial invertebrates, e.g., earthworms and isopods, presented a great potential to bioaccumulate PAHs from soils through dermal uptake and/or ingestion of underground water (Cachada et al. 2016; Kariyawasam et al. 2021). Terrestrial snails live at the interface soil-air and thus have been used as sentinel indicators in the evaluation of terrestrial pollution due to their wide environmental distribution and capacity to bioaccumulate pollutants (Al-Alam et al. 2019). Snails are exposed through the inhalation of airborne PAHs, ingestion of feed and water, and via cutaneous contact with contaminated soil (Louzon et al. 2020). Baroudi and co-authors compared, for the first time, two different biomonitors, terrestrial snails and tree leaves, in order to improve environmental biomonitoring assessment (Baroudi et al. 2021). Land snails presented a continuous increased concentration of PAHs during the sampling period (total PAHs: 1.09–44.15 ng/g) even during the precipitation season, while for pine needles, higher concentrations were observed in the beginning of the study (Baroudi et al. 2021). The PAHs’ levels decreased during the time (Baroudi et al. 2021). Terrestrial insects are very important species in several food webs, with some of them being vital pollinators. Levels of total PAHs reported in insects including honeybees ranged from 0.21 to 19,200 ng/g (Fig. 3 and Online Resource 11). The environmental contamination that affects insects will adversely affect the whole ecosystem (Al-Alam et al. 2019; Tan et al. 2018).

Honeybees are very sensitive organisms that present a vital role in environmental quality and diversity. These very important bioindicators move from plant to plant during regular forging activities and pollen transport across different plants and flowers. Levels of total PAHs reported in honeybees varied between 0.21 and 553 ng/g (Fig. 3 and Online Resource 11). The compounds BaP (0.058 to 2.45 ng/g), BaA (0.05–2.86 ng/g), Chry (0.04–1.98 ng/g), and DBalPyr (0.06–5.87 ng/g) have also been detected in the tissues of honeybees. Contamination of honeybees with ambient pollutants might occur through direct contact with contaminated air, soil, and water during beekeeping practices (Al-Alam et al. 2019). Honeybees are efficient sentinels of environmental contamination within a radius ranging from 1.5 to 3 km around the hive and carry pollutants including PAHs back to their hives and accumulate the contaminants in the brood and further on different honeybees’ by-products (e.g., honey, pollen, wax, and propolis) (Al-Alam et al. 2019; Badiou-Bénéteau et al. 2013; Kargar et al. 2017). Therefore, biomonitoring of honeybees allows long-term surveillance of the colony site and surrounding areas, enabling the study of ecotoxicology gradients over space and time in a selected geographical area (Cochard et al. 2021; Cunningham et al. 2022; Lambert et al. 2012).

Available biomonitoring studies performed with plants and animals undeniably demonstrate the dynamic interaction between the environmental contamination with PAHs, their incorporation in food chains, and consequent bioaccumulation on living species. Environmental biomonitoring studies can also be used to identify locations that need intervention and for a continuous follow-up of a specific place or species at risk, as well as to evaluate the impact of implemented policy actions. These studies can and should be used in the definition of critical ambient levels, thus contributing to a progressive improvement of environmental quality, protection, and preservation of biodiversity. Environmental biomonitoring and surveillance will promote more sustainable development and contribute to achieving the goals of the European Green Deal (European Comission 2019b) as well as the expected socioecological equilibrium between human-animal-environment interaction recommended by WHO-OHHLEP.

##### Human exposure to PAHs

Human biomonitoring assesses the total internal dose of PAHs, regardless the route of exposure (Angerer et al. 2007; Choi et al. 2017; Gao et al. 2018). Regarding PAHs, different biomarkers of exposure have been considered in HBM, namely unmetabolized and monohydroxylated PAHs in the urine, saliva, and breast milk (Alshaarawy et al. 2013; Barros et al. 2023b; Hwang et al. 2022; Ifegwu and Anyakora 2015; Oliveira et al. 2019, 2020c; Torres-Moreno et al. 2022). Analytical techniques to measure biomarkers in different biological fluids have been progressively advancing, allowing the detection of a wide range of biomarkers at ever-lower concentrations (Santos et al. 2019; Tan et al. 2012).

HBM have been widely used to characterize human exposures to PAHs due to the interaction with environmental media and during regular working activities (Barros et al. 2021; Engelsman et al. 2020; Louro et al. 2019; Oliveira et al. 2019, 2021; Pena et al. 2022). Based on data collected from different epidemiologic studies, IARC classified some occupational activities as known carcinogenic to humans (e.g., activities related to coal gasification, coke production, coal-tar distillation, chimney sweeping, paving, and roofing with coal-tar pitch, work with mineral oils, shale-oil, and aluminum production, activity as a firefighter, among others) (Barros et al. 2021; Demers et al. 2022; Ekpe et al. 2021; Engelsman et al. 2020; IARC 2010a; Jameson 2019; Oliveira et al. 2020a). Workers from these occupations are regularly exposed to increased levels of PAHs when compared with other occupations (e.g., administrative personnel) and the results from epidemiological studies demonstrated a higher incidence of occupational cancers (Andersen et al. 2021; Jalilian et al. 2019; Jameson 2019; Rehman et al. 2020; Soteriades et al. 2019). Moreover, other occupational activities related to transport (Andersen et al. 2021), cooking (Oliveira et al. 2021; Samir et al. 2019), and industrial workers (Orru et al. 2020; Sartorelli et al. 2020) are also regularly exposed to PAHs.

HBM also characterizes populations’ exposure to PAHs, particularly the most vulnerable groups such as children, pregnant women, and elderly (Best et al. 2016; Choi et al. 2017; Drwal et al. 2019; Hisamuddin and Jalaludin 2022; Oliveira et al. 2019, 2020c). However, the number of literature reviews addressing the characterization of general population exposure to PAHs via HBM remains limited. Studies performed in cord blood and placenta demonstrated fetus’ exposure to PAHs with values of total low-molecular weight PAHs of 20,621 ng/mL and 603 ng/g, respectively (Drwal et al. 2019; Rezaei Kalantary et al. 2020). Also, available studies found PAHs and/or PAH metabolites in the blood (0.04–76.54 ng/mL), milk (0.09–67.9 ng/mL), and urine (16–383 ng/mL) of nursing mothers (Drwal et al. 2019; Fernández et al. 2021; Khanverdiluo et al. 2021; Oliveira et al. 2020c; Yang et al. 2020). Moreover, Urbancova et al. (2020) reported a higher intake of PAHs in new-borns whose mothers live in more polluted areas than in less industrialized areas by presenting increased concentrations of total PAH metabolites (9.28 versus 4.92 μg/g creatinine). Oliveira et al. (2020c) reported the levels of total PAHs and PAH metabolites ranging between 55.2 and 1119 ng/g fat and from 6.66 to 455 ng/g fat in the breast milk of Portuguese nursing mothers. Those authors found increased values of PAHs in nursing mothers with more than 30 years and in those whose child was born with a lower weight. BaP and its main metabolite, 3-hydroxybenzo(a)pyrene, were not detected in the breast milk samples.

Concerning adults, several HBM have been performed to assess exposure to PAHs through the evaluation of exposure biomarkers (Choi et al. 2017; Hoseini et al. 2018; Huang et al. 2022; Iamiceli et al. 2020; Keir et al. 2021; Ratelle et al. 2020; Thai et al. 2020). HBM are so important for human health surveillance that several initiatives have been conducted in different countries. Since the legislative authorization in 1956, the USA designed and is continuously improving the program for the National Health And Nutrition Examination Survey (NHANES), which routinely provides information about the amount, distribution, and effects of illness and disability as well as on nutritional status among the American population (Best et al. 2016; Shiue 2015). This regular human biomonitoring survey includes the determination of different biomarkers of exposure to PAHs, and urinary monohydroxy-PAH metabolites in children and adults. Recently, some authors reported the levels of 1-hydroxynaphthalene (104 and 12,515,700 ng/g), 2-hydroxynaphthalene (222.83 and 317,670 ng/g), 2-hydroxyfluorene (16.67–27,889 ng/g), 1-hydroxyphenanthrene (3.70–19,497.20 ng/g), and 1-hydroxyprene (2.92–11,554.90 ng/g) among the American adults population (Wang et al. 2022a). Moreover, it was demonstrated a positive association between urinary PAH metabolites, diabetes mellitus (Mallah et al. 2022a), and the risk of hypertension among the American population (Mallah et al. 2022a; Wang et al. 2022a). Similarly, the Canadian Health Measures Survey (CHMS) is an initiative that provides biomonitoring data from the general population (adults and children) and also includes information about exposure to PAHs (Haines et al. 2017). The predominant metabolites of PAHs found in the Canadian population were 2-hydroxynaphthalene (3.80–4.40 µg/g creatinine), 1-hydroxynaphthalene (0.78–0.99 µg/g creatinine), 2-hydroxyfluorene (0.22–0.28 µg/g creatinine), 9-hydroxyfluorene (0.12–0.15 µg/g creatinine), 1-hydroxyphenanthrene (0.13–0.16 µg/g creatinine), and 1-hydroxypyrene (0.099–0.12 µg/g creatinine) (Health Canada 2017). This aspect has also been investigated in the Australian population being the reported concentrations similar to the levels found in developed countries and lower than the values observed at developing countries (Thai et al. 2020). Despite the fast development and industrialization observed in Southeast Asia, limited national programs of HBM have been implemented. Barnett-Itzhaki et al. (2018) emphasized the importance of results from HBM in environmental health policy as well as the need for harmonized protocols. In 2017/2018 started the first cohort study performed with the Chinese population, China National Human Biomonitoring (CNHBM), being included the determination of 9 PAH metabolites (Cao et al. 2021). Huang et al. (2022) also evaluated the presence of monohydroxylated PAHs in the population of different Chinese cities and reported more significant exposure to PAH in males, overweight people, and adults aged 18–59 years compared to children and elderly people. Median urinary concentrations were reported for 2-hydroxynaphthalene (4.75 µg/L), 1-hydroxynaphthalene (1.82 µg/L), 3-hydroxyfluorene (1.23 µg/L), 2-hydroxyfluorene (1.06 µg/L), 4-hydroxyphenanthrene (0.22 µg/L), and 1-hydroxypyrene (0.29 µg/L) (Huang et al. 2022). Regarding Europe, a European Human Biomonitoring Initiative was conducted between 2017 and 2021 (HBM4EU). A study conducted in 2020, with 300 adults from Switzerland, reported median levels of 1-hydroxynaphthalene, 1-hydroxypyrene, and 2-hydroxynaphthalene of 0.12 µg/L, 0.07 µg/L, and 2.76 µg/L, respectively (European Comission 2021). Louro et al. (2019) reviewed different HBM studies performed with European citizens and emphasized the importance of including HBM in future regulatory risk assessment as well as the combination of these studies with in vitro and in silico data. Those authors also highlighted the need for a legal effort to implement biological limit values in European Union and it was suggested to introduce HBM data in legislative risk assessment (Louro et al. 2019). Relatively to Portugal, Pena et al. (2022) reviewed the results obtained by Portuguese HBM studies and reported the main metabolites of low molecular weight PAHs as the most abundant biomarkers of exposure to PAHs [1-hydroxynaphthalene + 1-hydroxyacenaphthene (0.051–14.4 µmol/mol creatinine), 2-hydroxyfluorene (1.24 × 10^−4^–1.34 µmol/mol creatinine), 1-hydroxyphenanthrene (1.06 × 10^−2^–0.301 µmol/mol creatinine), and 1-hydroxypyrene (1.84 × 10^−3^–0.941 µmol/mol creatinine)]. In 2009, the BIOAMBIENT.ES project was promoted to estimate reference levels of environmental pollutants with samples collected from Spanish adults (Bartolomé et al. 2015). For example, for lactating mothers, the predominant urinary metabolites of PAHs were 2-hydroxynaphthalene (7.1 ng/mL) and 1-hydroxynaphthalene (0.8 ng/mL), which is aligned with the data retrieved from other HBM studies (F. Fernández et al. 2021). Overall, the reported concentrations of urinary PAH metabolites in Spanish adults were higher than those found in the North American population but in close range and sometimes lower than the levels found in other European countries (Bartolomé et al. 2015). The German Environmental Survey for Children and Adolescents (GerES V) was conducted between 2014 and 2017 and described higher concentrations of PAH metabolites in young children compared to adolescents (0.379 versus 0.437 µg/L), and also in the population from East Germany compared to those living in West Germany (0.471 versus 0.372 µg/L) (Murawski et al. 2020). The predominant metabolites were 2-hydroxynaphthalene (3.706 μg/g creatinine), 1-hydroxynaphthalene (0.688 μg/g creatinine), and the metabolites of Phen (0.448 μg/g creatinine) (Murawski et al. 2020). The MATCH project conducted among the UK population evaluated the PAH exposure in non-smoker adults without relevant occupational exposure (Aquilina et al. 2010). The research team found a correlation between atmospheric exposures to higher molecular weight PAHs and the concentrations of urinary nicotine metabolites, thus demonstrating the exposure to environmental tobacco smoke as an important source of PAHs to non-smokers (Aquilina et al. 2010). Data related to human exposure to PAHs via HBM studies are difficult to compare because the concentrations of urinary PAH metabolites are frequently not adequately normalized with personal creatinine levels, which allow accounting for individual variability (e.g., fluid intake, physical exercise, and body temperature).

Environmental contamination is directly related to human exposure to PAHs and associated health risks. More regular HBM should be promoted to better characterize human exposure to PAHs, taking into consideration different age groups and different occupational activities. Additionally, there is an urgent need to define reference values for the predominant urinary PAH metabolites which will be determinant for the interpretation of biological data and future preventive/mitigation actions to promote human health. The potential of combining biomarkers of exposure and effect to provide a more comprehensive analysis of possible health risks should also be further explored. This approach could be helpful for identification of relationships between dysfunctions in biological mechanisms that can trigger the development of certain diseases and exposure to PAHs. This association can be useful to recognize the context and the type of environment that can contribute to these adverse effects (Barros et al. 2021, 2023a, 2023b).

## Health effects

Available literature clear demonstrates the ubiquity of PAHs throughout different environmental media and their contribution to the worldwide chemical pollution crisis. Human exposure to PAHs occurs through inhalation, ingestion, and dermal contact and represents an additional risk for human health, due to the carcinogenic, teratogenic, and mutagenic properties of some PAHs (Fig. 1) (Famiyeh et al. 2021; Gao et al. 2018; Kamal et al. 2015; Mallah et al. 2022c; Sun et al. 2021; WHO 2013; Zhang et al. 2022a). Potential health risks are dependent on the route and duration of exposure as well as on the composition, concentrations, and toxicity of the mixture of PAHs (Patel et al. 2020). Only the bioavailable form of the xenobiotic is assimilated in tissues and body fluids (Ifegwu and Anyakora 2015). After absorption into the human body, PAHs reach the lymph and circulate in the blood, being distributed among all tissues and organs. PAHs are primarily metabolized in the liver and kidney, and to a less extent in the lungs, intestinal mucosa, and skin (WHO 2000). The metabolization of PAHs is mediated by the enzymatic family of cytochrome P450 to facilitate their excretion through biological fluids (e.g., bile, feces, urine, sweat, and milk) (Angerer et al. 2007; Gao et al. 2018; Vogel et al. 2020). The toxicological potential of PAHs is associated with the generation of reactive electrophilic intermediates such as conjugated hydroxyl-alkyl derivatives, quinones and diol-epoxides, during the metabolization process (WHO 2000). Genotoxicity of PAHs is related to the ability of the generated intermediates to induce the synthesis of reactive oxygen species that promote mutations in proto-oncogenes and tumor suppressor genes (Jameson 2019). Carcinogenicity of PAHs is caused by the development of a site-specific tumor at the human body (da Silva Junior et al. 2021; Sun et al. 2021; Zhang et al. 2021).

Among exposed populations, children, pregnant/nursing women, people with pre-existing health disorders, and the elderly raise more concern due to their higher susceptibility to the potential health risks caused and/or aggravated by regular exposure to PAHs (Castagna et al. 2022; Oliveira et al. 2019). Health problems related to poor air quality include respiratory and cardiovascular diseases, asthma, and allergy, being considered a very serious problem by European citizens (European Comission 2019a). Air pollution, including PM_2.5_, is one of the main causes of premature death and it is responsible for around 400,000 premature deaths per year among European citizens (excluding the Turkish) (World Health Assembly 2018). Also, the presence of PAHs and other health-relevant pollutants adsorbed/absorbed on PM also contributes to the health risks associated with exposure to particles being well described in the literature (Kim et al. 2013; Landrigan et al. 2018; Oliveira et al. 2015, 2019). Heart disease and stroke are the most common causes of premature deaths attributed to air pollution, followed by lung diseases including cancer (Elonheimo et al. 2022; Shahriyari et al. 2022; Xue et al. 2022; World Health Assembly 2018). Maternal exposure to air pollution is also associated with adverse effects on fertility and pregnancy as well as on the health of newborns and children (Veber et al. 2022; WHO 2013). There is also emerging evidence that exposure to air pollution is associated with new‑onset type 2 diabetes (Khosravipour and Khosravipour 2020; Wang et al. 2022b) in adults and it may be also linked with obesity (Bushnik et al. 2020; Shi et al. 2022), systemic inflammation (Alshaarawy et al. 2013; Mohammadi et al. 2022), Alzheimer’s disease, and dementia (Best et al. 2016). Exposure to airborne PAHs has been associated with the formation of oxidative stress species and the development of inflammation processes (Xue et al. 2022), and increased cancer incidence (e.g., breast and lung cancers) (WHO 2021). Epidemiological studies have shown that PAHs are associated with reduced lung function, exacerbation of asthma, and increased rates of obstructive lung and cardiovascular diseases (WHO 2021). Several works already associated exposure to air pollutants, including but not limited to PAHs, with reducing productivity due to working days lost and reduction in life expectancy with considerably increased medical costs and high economic impact on societies (EEA 2020).

Limited epidemiological evidence suggests that exposure to airborne PAHs can lead to adverse effects on cognitive or behavioral function in children (WHO 2021). Drwal et al. (2019) reviewed different HBM studies concerning the role of placental function on prenatal exposure to PAHs, and it was demonstrated a higher incidence of intrauterine growth retardation, developmental toxicity, and cancer risk due to susceptibility to DNA damage, low intelligence quotient, and a higher predisposition to develop allergies and asthma in infancy. Indeed, children are more sensitive than adults because they present higher absorption rates through inhalation and dermal contact due to different physiology, metabolism, and social behaviors (Oliveira et al. 2019). Compared to adults, the half-life time of xenobiotics in children’s bodies could be longer since they present reduced body size and blood volume capacity as well as less mature metabolic and immunological systems (Choi et al. 2017). Therefore, exposure to PAHs during infancy may have implications on children’s development and on their health at the adult age (Bushnik et al. 2020; Castagna et al. 2022; Choi et al. 2017; Díaz de León-Martínez et al. 2021; Fernández et al. 2021; Murawski et al. 2020; Oliveira et al. 2019). Moreover, positive correlations have been reported between biomarkers of exposure to PAHs (PAH metabolites) and genotoxic endpoints, adducts of BaP, PAH–protein adducts, PAH–DNA adducts, and biomarkers of inflammation (Alshaarawy et al. 2013; Barros et al. 2021; Hwang et al. 2022; Ifegwu and Anyakora 2015; Oliveira et al. 2019, 2020c; Torres-Moreno et al. 2022). Despite the different health risks associated with human exposure to PAHs, data related with the key-driving mechanisms remains scarce and should be explored in future studies. Also, the antagonist and/or synergetic relation between PAHs and other persistent organic pollutants and their contribution to human health burden need to be characterized.

## Concluding remarks

A broad and comprehensive description of PAHs’ ubiquity, accounting for environmental contamination and biomonitoring assays, is for the first time presented. Environmental contamination with PAHs contributes to the global chemical pollution crisis, which is the key driver of the dynamic system between the inextricably interconnected environmental and human health. The presence of PAHs in different environmental matrices is clearly demonstrated, raising concern among the scientific community and governmental authorities on their hazardous effects. The higher rates of human dependency on pollutant anthropogenic activities (e.g., petroleum derivatives, energy production, residential heating and cooking, transports) are directly associated with environmental contamination with PAHs (1344.4–12,300 ng/m^3^ versus 0.03–0.60 ng/m^3^ in the air of industrial/urban and rural areas, respectively; 2.00–1.66 × 10^7^ ng/L and 7.00 × 10^−4^–1.00 × 10^9^ ng/g in the water systems and aquatic sediments from coastal areas, respectively; 0.14–1.77 × 10^6^ ng/g and 1.59–5.87 × 10^3^ ng/g in the urban and rural soils, respectively) and their bioaccumulation in different aquatic and terrestrial plants and animals, principally in the coastal and more urbanized/industrialized areas. Different environmental biomonitoring studies have identified some sentinel species (e.g., mosses, lichens, cephalopods, snails, and honeybees) as environmental biomonitors able to characterize the contamination with PAHs in their surrounding media. Despite the great effort to reduce the strong human dependence on fossil fuels and coal, it is expected on the next decades an important dependence which will contribute to increase the environmental contamination with PAHs. Additionally, climate change and global warming progressively increase the intensity and severity of wildfires which represent a global emission source of PAHs, among other pollutants, with important implications for the health of ecosystems as well as for human lives.

Available literature highlights the need to mitigate the presence of PAHs in different environmental media, principally in the most industrial and urbanized areas. Therefore, future research should pursue the development and implementation of effective (bio)remediation tools to reduce/mitigate the bioaccumulation of PAHs in the air, water resources, and soils with consequent assimilation on different trophic levels which will contribute to minimizing the risks for the environment and the human health. The phytoextraction with different species of plants (Sawicka et al. 2022), the use of biochars (Valizadeh et al. 2022; Zhang et al. 2022c), and application of biosurfactants produced by microorganisms (Cazals et al. 2022; Vaidyanathan et al. 2022; Zhang et al. 2022b) are some of the (bio)remediation tools that are already under evaluation to reduce the contamination of soils. Concerning water resources, biological treatment methods (e.g., bioreactors, phytoremediation, bioremediation, activated sludge process, sequencing batch reactors, and membrane bioreactors) and physicochemical methods (e.g., membrane filtration, adsorption, advanced oxidation processes, and chemical precipitation) have been applied (Gutierrez-Urbano et al. 2021). Reduction of air pollutant emissions has been the main target of the European Green Deal program which aims to reach carbon neutrality by 2050. Some of the mitigation measures include the preference for renewable energies (nor fossil or nuclear fuels), reduction of automobile traffic, investment in public transport, and renovation of buildings to be more energy efficient (Chen et al. 2022; Wolf et al. 2021).

Literature demonstrated the great potential of HBM to characterize occupational and environmental exposure to PAHs and should be used in the near future for the definition of critical levels of human exposure. Around the world, different initiatives are being conducted to characterize exposure to PAHs in different occupations and age groups of the population; however, most of the available surveillance programs are discontinuous and do not represent all the population groups. Under the climate change, global warming scenario, and the chemical pollution crisis, the environmental and human exposure to PAHs is expected to increase, which represents an additional risk for environmental contamination and human health. Taking into account the One Health approach recommended by WHO, mitigation and preventive measures for PAH exposure can be beneficial to protect human health due to the improvement in the human-animal-environment interface (WHO 2022). Human health is interconnected with animal and environmental health since pollutants such as PAHs are accumulated in animals and plants which will be consumed by humans with demonstrated adverse health effects (WHO 2022). Therefore, more studies producing additional knowledge on environmental contamination and human exposure to PAHs would be crucial to improve the characterization of the negative effects of PAHs on environmental media, biota, and on human health. Environmental biomonitoring and HBM studies can also contribute to the implementation and development of more sustainable policies that can contribute to the execution and concretization of the European Green Deal Programme and pursue the recommended mobilization of transdisciplinary and multisectoral collaboration between scientific, social, technologic, and industrial sectors toward the One-Health Perspective desired by WHO-OHHLEP. The results of these studies will support governmental authorities in the implementation of regulatory guidelines and protective measures for the environment and the preservation of biodiversity with direct consequences for human health and the quality of life.

## Supplementary Information

Below is the link to the electronic supplementary material.

Supplementary file1 (DOCX 170 KB)

Supplementary file2 (DOCX 122 KB)

Supplementary file3 (DOCX 33 KB)

Supplementary file4 (DOCX 335 KB)

Supplementary file5 (DOCX 211 KB)

Supplementary file6 (DOCX 31 KB)

Supplementary file7 (DOCX 227 KB)

Supplementary file8 (DOCX 53 KB)

Supplementary file9 (DOCX 201 KB)

Supplementary file10 (DOCX 161 KB)

Supplementary file11 (DOCX 124 KB)

## Data Availability

Data collected from available literature and analyzed during this work are included in this research paper and Supplementary Material. However, extracted raw data can be made available on reasonable request after formal approval from the host institution.
