# Supplementary material for: Environmental contamination with polycyclic aromatic hydrocarbons and contribution from biomonitoring studies to the surveillance of global health
Source: Environ Sci Pollut Res Int. 2024 Aug 29;31(42):54339–62. doi: 10.1007/s11356-024-34727-3 (PMC11413127; doi:10.1007/s11356-024-34727-3)
Supplement: Supplementary file 1 — Supplementary file1 (DOCX 170 KB) [file 11356_2024_34727_MOESM1_ESM.docx]

**Online Resource 1**

Environmental contamination with polycyclic aromatic hydrocarbons and contribution from biomonitoring studies to the surveillance of global health

Joana Teixeira, Cristina Delerue-Matos, Simone Morais, Marta Oliveira*

REQUIMTE/LAQV, ISEP, Polytechnique of Porto, Rua Dr. António Bernardino de Almeida 431, 4249-015, Porto, Portugal

*Corresponding author: Tel.: +351 22 834 0500

E-mail: *marta.oliveira@graq.isep.ipp.pt*

List of 16 PAHs included in the priority list of pollutants and physicochemical properties.

| PAH | Structure (Formula) | | No. of rings | | Molecular weight  (g/mol) | | Vapor pressure  (Pa at 25 ^o^C) | | Boiling point  (^o^C) | | Melting point  (^o^C) | | Solubility  (mg/L) | |
| --- | --- | --- | --- | --- | --- | --- | --- | --- | --- | --- | --- | --- | --- | --- |
| Naphthalene | 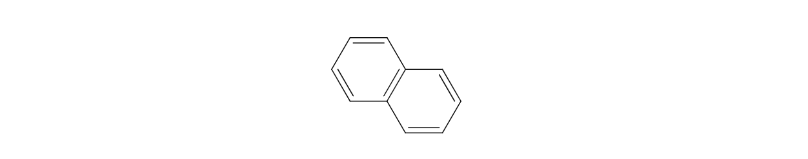(C_10_H_8_) | | 2 | | 128 | | 11.9 | | 218 | | 80 | | 30.0 | |
| Acenaphthylene | 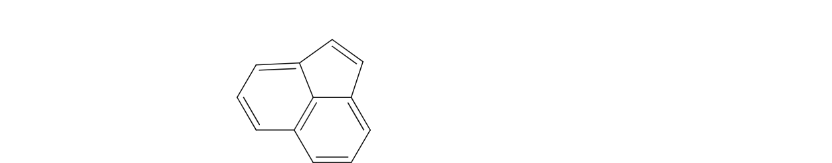(C_12_H_8_) | | 3 | | 152 | | 3.86 | | 280 | | 124 | | 3.93 | |
| Acenaphthene | 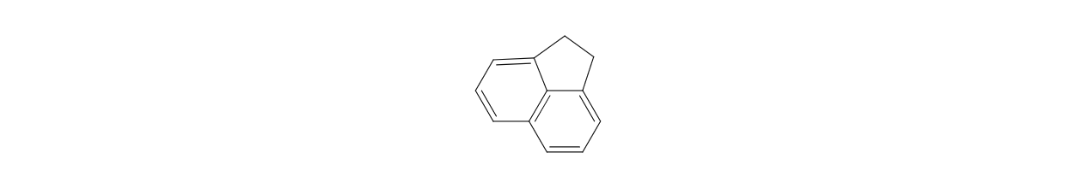(C_12_H_10_) | | 3 | | 154 | | 0.50 | | 279 | | 108 | | 3.93 | |
| Fluorene | 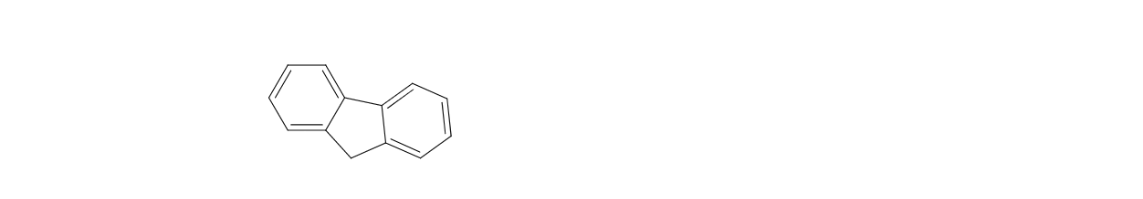 (C_13_H_10_) | | 3 | | 166 | | 0.43 | | 295 | | 119 | | 1.98 | |
| Phenanthrene | 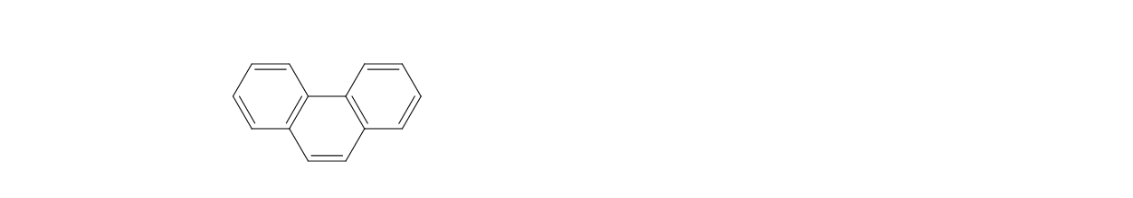 (C_14_H_10_) | | 3 | | 178 | | 9.1 × 10^-2^ | | 340 | | 100 | | 1.15 | |
| Anthracene | 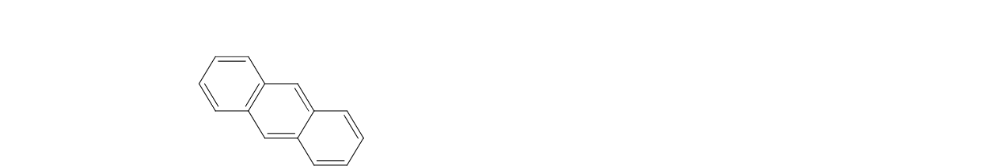(C_14_H_10_) | | 3 | | 178 | | 3.4 × 10^-3^ | | 342 | | 216 | | 0.015 | |
| Fluoranthene | 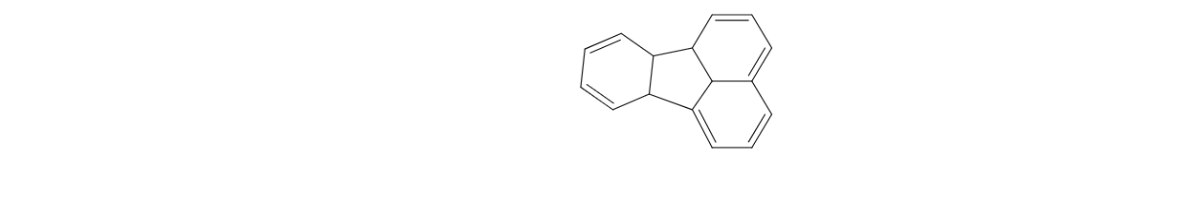(C_16_H_10_) | | 4 | | 202 | | 1.2 × 10^-3^ | | 375 | | 109 | | 0.25 | |
| Pyrene | 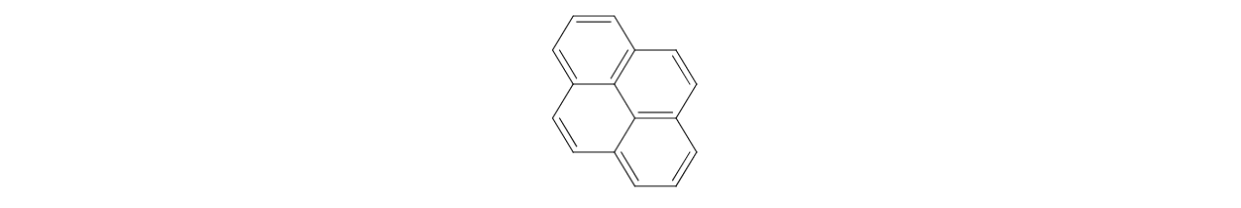(C_16_H_10_) | | 4 | | 202 | | 6.0 × 10^-4^ | | 393 | | 150 | | 0.132 | |
| Chrysene | 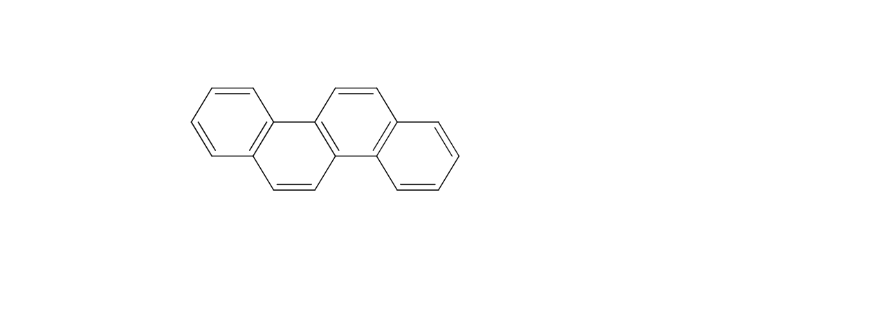 (C_18_H_12_) | | 4 | | 228 | | 1.0 × 10^-6^ | | 400 | | 228 | | 0.002 | |
| Benz(a)anthracene | | | 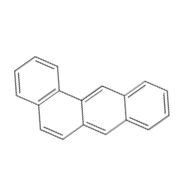(C_18_H_12_) | | 4 | | 228 | | 1.5 × 10^-4^ | | 438 | 162 | 0.009 | |
| Benzo(b)fluoranthene | | | 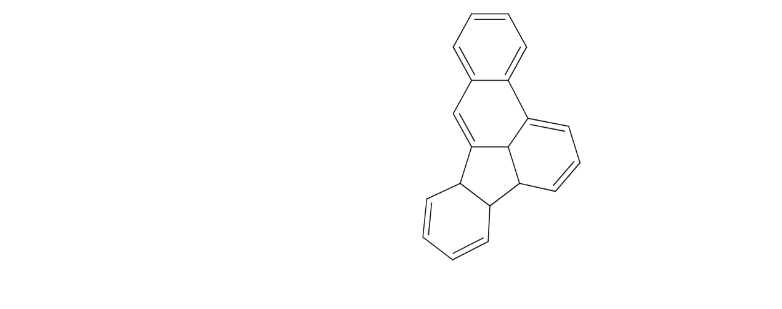 (C_20_H_12_) | | 5 | | 252 | | 6.7 × 10^-5^ | | 481 | 168.3 | 0.002 | |
| Benzo(k)fluoranthene | | | 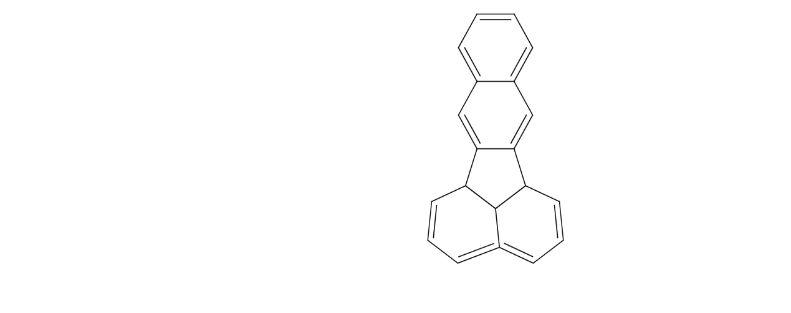(C_20_H_12_) | | 5 | | 252 | | 5.2 × 10^-8^ | | 480 | 215.7 | 0.0008 | |
| Benzo(a)pyrene | | | 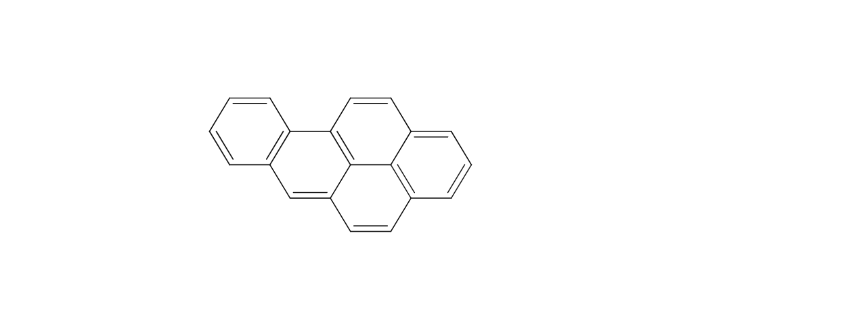 (C_20_H_12_) | | 5 | | 252 | | 0.4 × 10^-6^ | | 495 | 179 | 0.0038 | |
| Dibenz(a,h)anthracene | | | 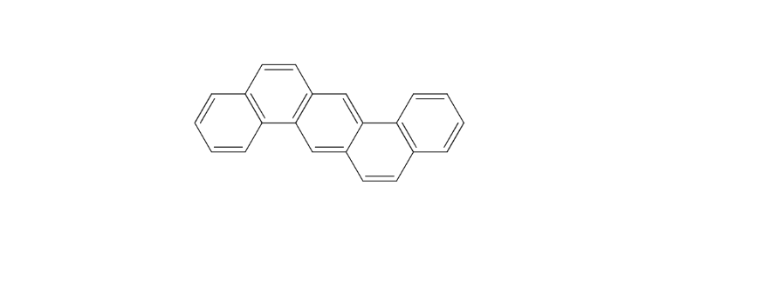 (C_22_H_14_) | | 5 | | 278 | | 2.8 × 10^-9^ | | 487 | 218 | 31.5 | |
| Benzo(g,h,i)perylene | | | 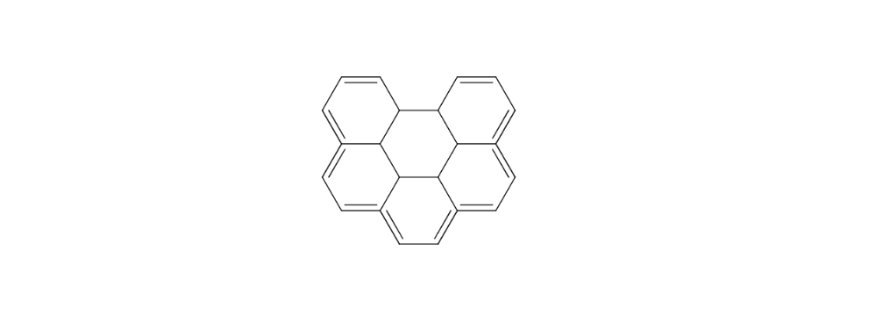 (C_22_H_12_) | | 6 | | 276 | | 6.0 × 10^-8^ | | 500 | 277 | 3.93 | |
| Indeno(1,2,3-c,d)pyrene | | | 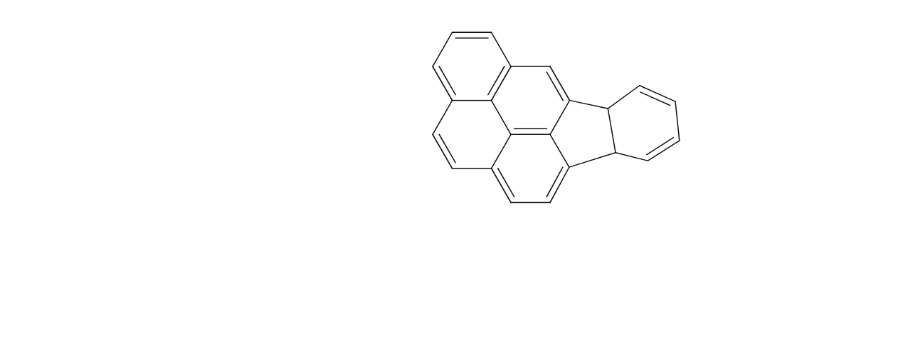(C_22_H_12_) | | 6 | | 276 | | 1.0 × 10^-10^ | | 498 | 233 | 0.0005 | |

Adapted from Sun *et al.* [26], Copyright (2023), with permission from Elsevier.
