# Supplementary material for: Environmental contamination with polycyclic aromatic hydrocarbons and contribution from biomonitoring studies to the surveillance of global health
Source: Environ Sci Pollut Res Int. 2024 Aug 29;31(42):54339–62. doi: 10.1007/s11356-024-34727-3 (PMC11413127; doi:10.1007/s11356-024-34727-3)
Supplement: Supplementary file 2 — Supplementary file2 (DOCX 122 KB) [file 11356_2024_34727_MOESM2_ESM.docx]

**Online Resource 2**

Environmental contamination with polycyclic aromatic hydrocarbons and contribution from biomonitoring studies to the surveillance of global health

Joana Teixeira, Cristina Delerue-Matos, Simone Morais, Marta Oliveira*

REQUIMTE/LAQV, ISEP, Polytechnique of Porto, Rua Dr. António Bernardino de Almeida 431, 4249-015, Porto, Portugal

*Corresponding author: Tel.: +351 22 834 0500

E-mail: *marta.oliveira@graq.isep.ipp.pt*


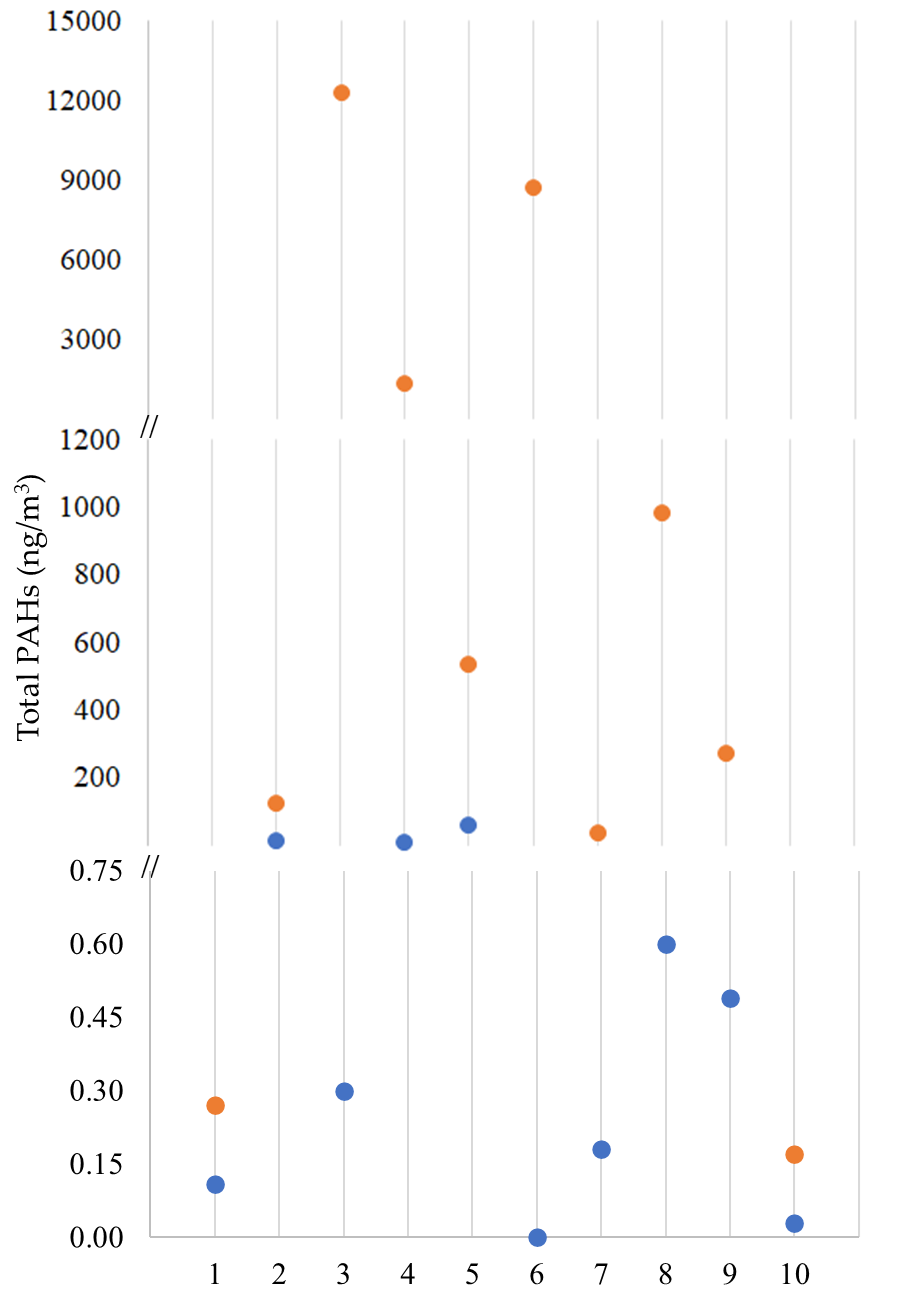


Levels of total PAHs (minimum – maximum, represented as blue and orange dots, respectively) reported in the ambient air [1 – Rovira et al., 2021; 2 – Oliveira et al., 2019; 3 - Srogi et al., 2007; 4 - Manzetti et al., 2013; 5 - Wallace et al., 2020; 6 - Ofori et al., 2020; 7 - Alvi et al., 2018; 8 - Liu et al., 2022; 9 - Castagna et al., 2022; 10 - Al-Alam et al., 2019]
