# Supplementary material for: Environmental contamination with polycyclic aromatic hydrocarbons and contribution from biomonitoring studies to the surveillance of global health
Source: Environ Sci Pollut Res Int. 2024 Aug 29;31(42):54339–62. doi: 10.1007/s11356-024-34727-3 (PMC11413127; doi:10.1007/s11356-024-34727-3)
Supplement: Supplementary file 3 — Supplementary file3 (DOCX 33 KB) [file 11356_2024_34727_MOESM3_ESM.docx]

**Online Resource 3**

Environmental contamination with polycyclic aromatic hydrocarbons and contribution from biomonitoring studies to the surveillance of global health

Joana Teixeira, Cristina Delerue-Matos, Simone Morais, Marta Oliveira*

REQUIMTE/LAQV, ISEP, Polytechnique of Porto, Rua Dr. António Bernardino de Almeida 431, 4249-015, Porto, Portugal

*Corresponding author: Tel.: +351 22 834 0500

E-mail: *marta.oliveira@graq.isep.ipp.pt*

Levels of possible and/or probable carcinogenic PAHs (expressed as range) reported in the ambient air (ng/m^3^ unless indicated otherwise), superficial water (ng/L), and water sediments (ng/g).

| PAH | Air | | | Superficial water | | Sediments | | | | | |
| --- | --- | --- | --- | --- | --- | --- | --- | --- | --- | --- | --- |
|  | Manzetti *et al.*, 2013 | Alvi *et al.*, 2018 | Abas, A., 2021^a^ | Jesus *et al.*, 2022 | Honda *et al.*, 2020 | Du *et al.*, 2018 | Jesus *et al.*, 2022 | Honda *et al.*, 2020 | Mearns *et al.*, 2019 | Wright *et al.*, 2018 |  |
| Benz(a)anthracene | 0.02 - 1.95 | 0.01 - 0.26 | 5.00 - 32.0 | 2.0×10^-2^ – 2.30 × 10^5^ | 4.30 × 10^5^ | - | 0.10 - 43000 | 0.03 - 1598 | 0.91 - 275 | 12.0 - 44.0 |  |
| Benzo(a)pyrene | 0.05 - 1.60 | 0.01 - 0.50 | 10.0 - 15.0 | 1.0×10^-2^ - 1.31 × 10^3^ | 4.30 × 10^5^ | 0.02 - 0.06 | 0.10 - 64000 | 0.05 - 1607 | 0.37 - 78.3 | 35.0 - 51.0 |  |
| Benzo(b)fluoranthene | 2.95 - 6.55 | 0.01 - 0.32 | - | 3.0×10^-2^ – 0.68 × 10^3^ | 4.90 × 10^5^ | 0.04 - 0.11 | 0.40 - 54000 | - | 3.73 - 370 | - |  |
| Benzo(k)fluoranthene | - | 0.01 - 0.34 | - | 1.0×10^-2^ - 0.85 × 10^3^ | 7.40 × 10^5^ | 0.04 - 0.16 | 0.20 - 28000 | - | 0.72 - 115 | - |  |
| Benzo(b+k)fluoranthene | - | - | 13.0 - 55.0 | - | - | - | - | - | - | - |  |
| Benzo(b+j+k)fluoranthene | - | - | - | - | - | - | - | 0.13 - 2769 | - | 36.0 - 64.0 |  |
| Chrysene | 0.01 - 2.55 | 0.01 - 0.35 | 14.0 - 105 | - | 2.20 × 10^5^ | - | - | 0.10 - 1756 | 0.90 - 230 | 15.0 - 68.0 |  |
| Dibenz(a,h)anthracene | - | 6.0×10^-3^ - 0.13 | 13.0 - 57.0 | 1.0×10^-2^ – 0.77 × 10^3^ | 2.00 × 10^5^ | - | 0.10 - 9000 | 0.01 - 247 | 0.26 - 12.2 | - |  |
| Indeno(1,2,3-c,d)pyrene | - | 0.01 - 0.64 | 11.0 - 43.0 | 2.0×10^-2^ – 3.15 × 10^3^ | 2.00 × 10^5^ | - | 0.10 - 57000 | 0.09 - 1296 | - | - |  |
| Naphthalene | 0.05 - 0.25 | 0.01 - 34.3 | 62.0 - 231 | 1.0×10^-2^ – 1.08 × 10^3^ | 1.00 × 10^6^ | 0.05 - 2.27 | 1.10 - 781 | 2.00 - 17.0 | 2.65 - 34.8 | - |  |

^a^Values presented in ng/sample
