# Supplementary material for: Environmental contamination with polycyclic aromatic hydrocarbons and contribution from biomonitoring studies to the surveillance of global health
Source: Environ Sci Pollut Res Int. 2024 Aug 29;31(42):54339–62. doi: 10.1007/s11356-024-34727-3 (PMC11413127; doi:10.1007/s11356-024-34727-3)
Supplement: Supplementary file 4 — Supplementary file4 (DOCX 335 KB) [file 11356_2024_34727_MOESM4_ESM.docx]

**Online Resource 4**

Environmental contamination with polycyclic aromatic hydrocarbons and contribution from biomonitoring studies to the surveillance of global health

Joana Teixeira, Cristina Delerue-Matos, Simone Morais, Marta Oliveira*

REQUIMTE/LAQV, ISEP, Polytechnique of Porto, Rua Dr. António Bernardino de Almeida 431, 4249-015, Porto, Portugal

*Corresponding author: Tel.: +351 22 834 0500

E-mail: *marta.oliveira@graq.isep.ipp.pt*

| 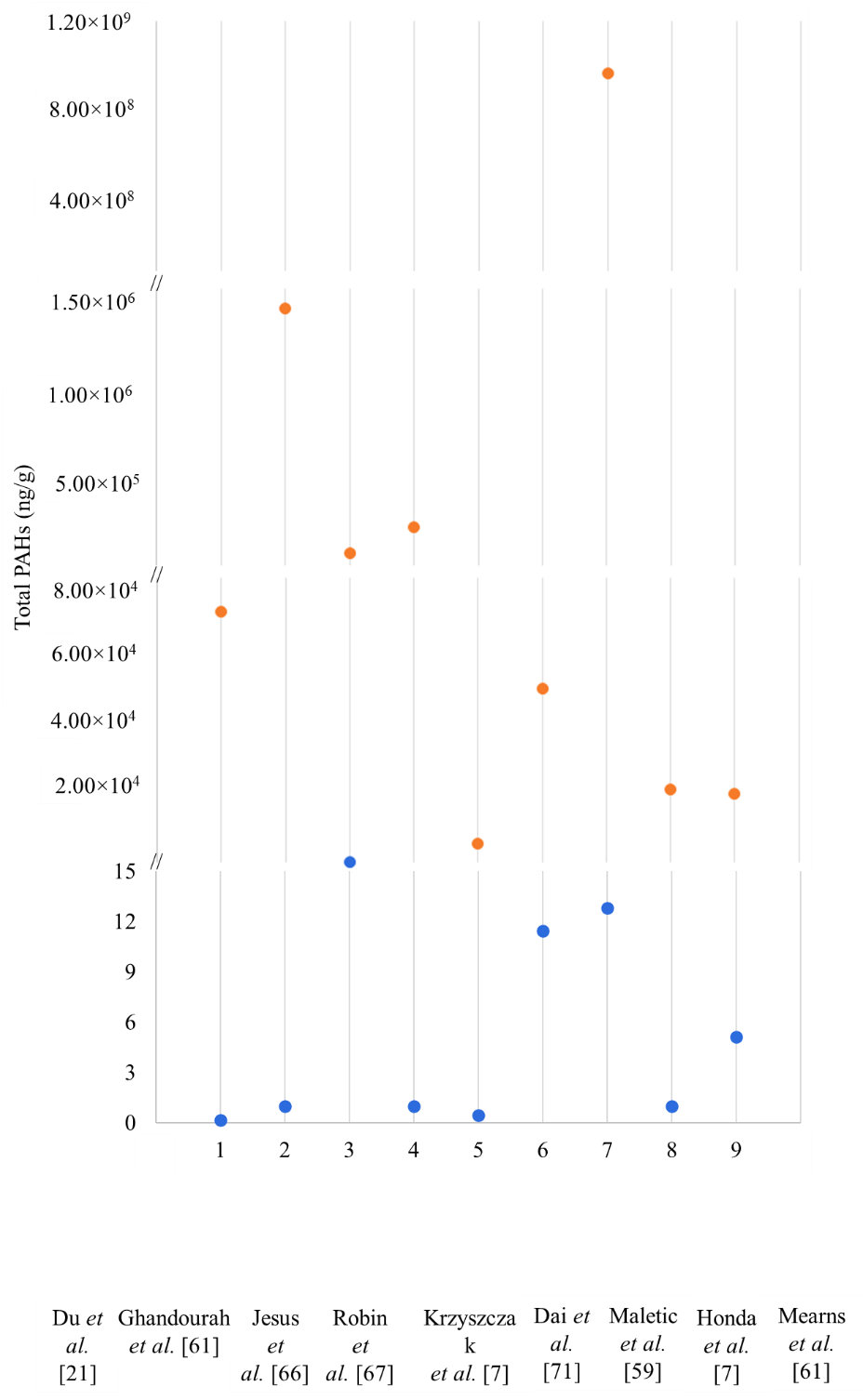 | 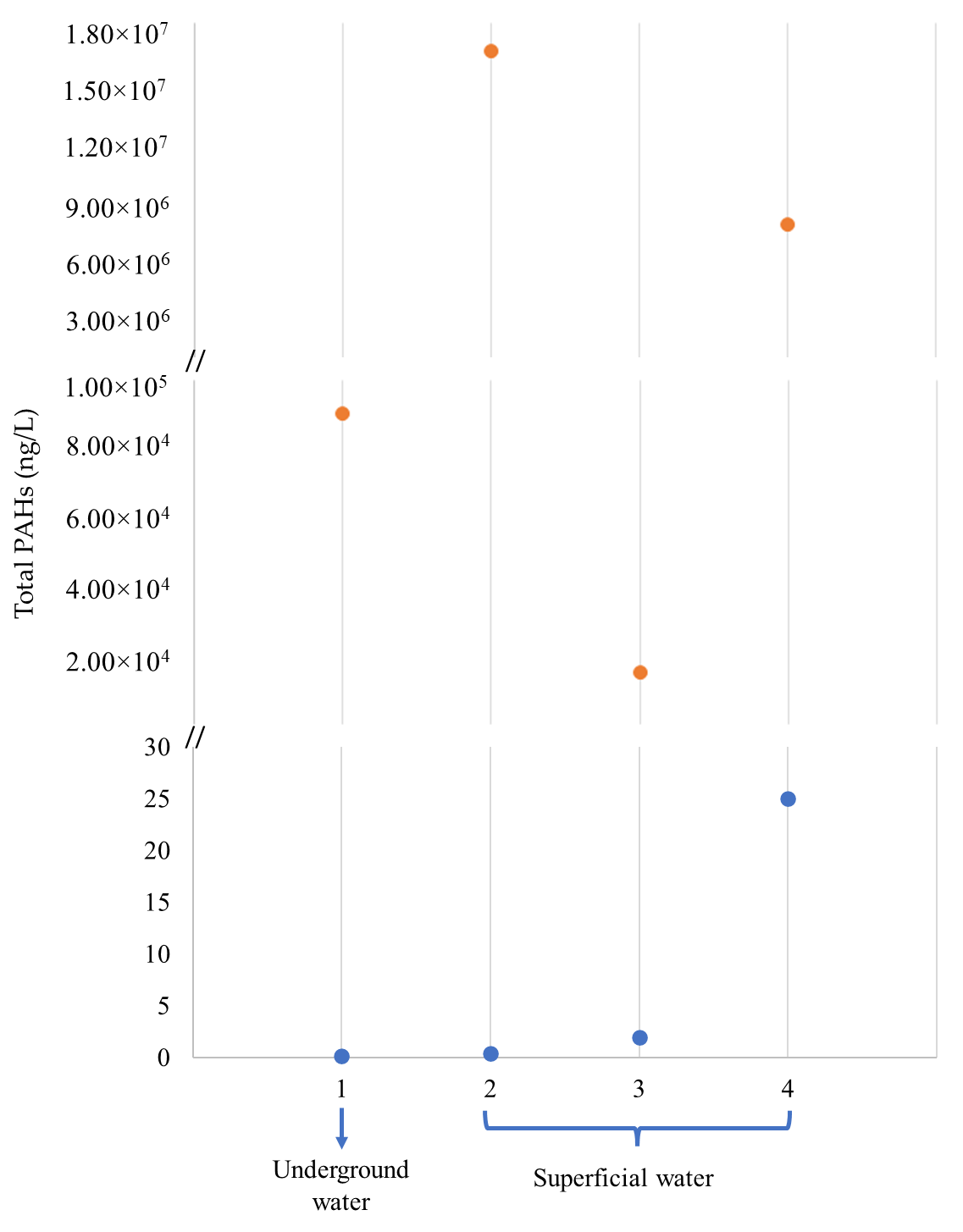 |
| --- | --- |
| a) | b) |

Levels of total PAHs (minimum – maximum, represented as blue and orange dots, respectively) reported in the a) aquatic sediments [1 – Du et al., 2018; 2 – Ghandourah et al., 2022; 3 - Jesus et al., 2022; 4 – Robin et al., 2022; 5 – Krzyszczak et al., 2021; 6 – Dai et al., 2022; 7 – Maletic et al., 2019; 8 – Honda et al., 2020; 9 – Mearns et al., 2019] and b) water systems [1 and 2 – Ofori et al., 2020; 3 – Jesus et al., 2022; 4 – Honda et al., 2020]
