# Supplementary material for: Environmental contamination with polycyclic aromatic hydrocarbons and contribution from biomonitoring studies to the surveillance of global health
Source: Environ Sci Pollut Res Int. 2024 Aug 29;31(42):54339–62. doi: 10.1007/s11356-024-34727-3 (PMC11413127; doi:10.1007/s11356-024-34727-3)
Supplement: Supplementary file 5 — Supplementary file5 (DOCX 211 KB) [file 11356_2024_34727_MOESM5_ESM.docx]

**Online Resource 5**

Environmental contamination with polycyclic aromatic hydrocarbons and contribution from biomonitoring studies to the surveillance of global health

Joana Teixeira, Cristina Delerue-Matos, Simone Morais, Marta Oliveira*

REQUIMTE/LAQV, ISEP, Polytechnique of Porto, Rua Dr. António Bernardino de Almeida 431, 4249-015, Porto, Portugal

*Corresponding author: Tel.: +351 22 834 0500

E-mail: *marta.oliveira@graq.isep.ipp.pt*

| 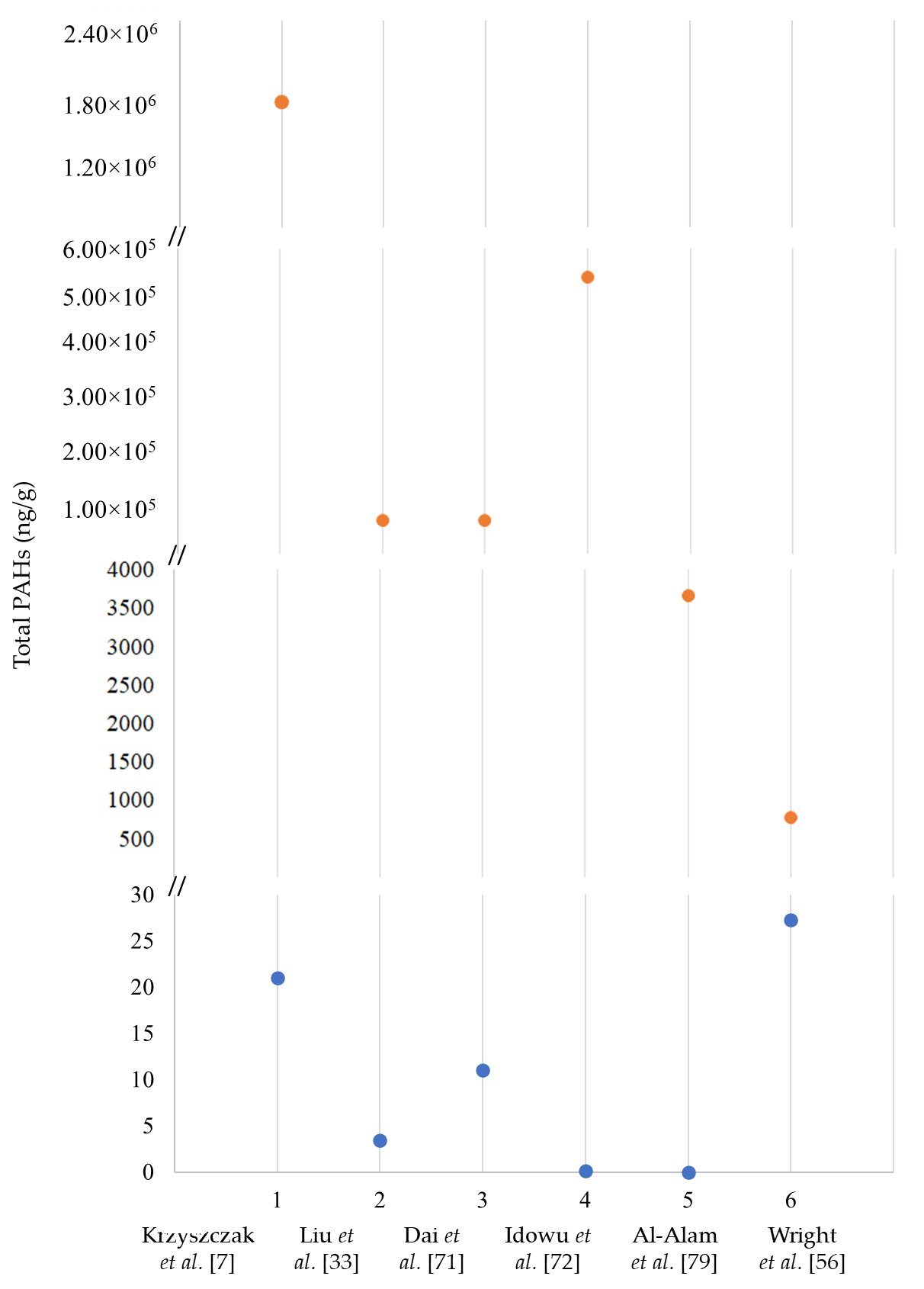 |
| --- |
| a) |
| 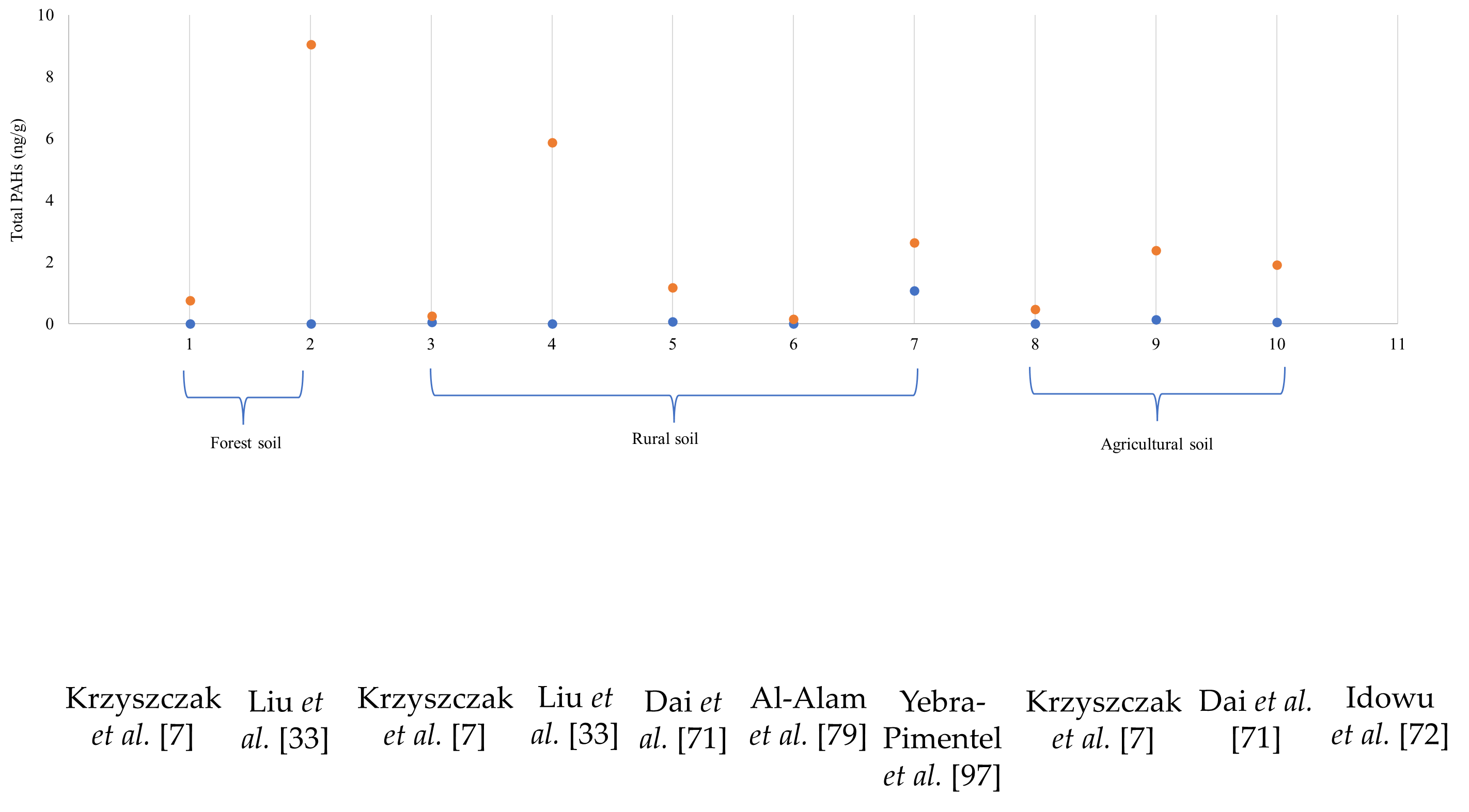 |
| b) |

Levels of total PAHs (minimum – maximum, represented as blue and orange dots, respectively) reported in a) urban soils [1 – Krzyszczak et al., 2021; 2 – Liu et al., 2022; 3 - Dai et al., 2022; 4 – Idowu et al., 2019; 5 – Al-Alam et al., 2019; 6 – Wright et al., 2021] and b) forest, rural and agricultural soils [1, 3, 8 - Krzyszczak et al., 2021; 2, 4 - Liu et al., 2022; 5, 9 – Dai et al., 2022; 6 – Al-Alam et al., 2019; 7 – Yebra-Pimentel et al., 2015; 10 – Idowu et al., 2019]
