# Supplementary material for: Environmental contamination with polycyclic aromatic hydrocarbons and contribution from biomonitoring studies to the surveillance of global health
Source: Environ Sci Pollut Res Int. 2024 Aug 29;31(42):54339–62. doi: 10.1007/s11356-024-34727-3 (PMC11413127; doi:10.1007/s11356-024-34727-3)
Supplement: Supplementary file 6 — Supplementary file6 (DOCX 31 KB) [file 11356_2024_34727_MOESM6_ESM.docx]

**Online Resource 6**

Environmental contamination with polycyclic aromatic hydrocarbons and contribution from biomonitoring studies to the surveillance of global health

Joana Teixeira, Cristina Delerue-Matos, Simone Morais, Marta Oliveira*

REQUIMTE/LAQV, ISEP, Polytechnique of Porto, Rua Dr. António Bernardino de Almeida 431, 4249-015, Porto, Portugal

*Corresponding author: Tel.: +351 22 834 0500

E-mail: *marta.oliveira@graq.isep.ipp.pt*

Levels of possible and/or probable carcinogenic PAHs (expressed as range; ng/g) reported in urban, forest and agricultural soils.

| PAH | Urban soil | Forest soil | | Agricultural soil |
| --- | --- | --- | --- | --- |
|  | Wright *et al.*, 2018 | Wright *et al.*, 2018 | Dolegowska *et al.*, 2021 | Yebra-Pimentel *et al.*, 2015 |
| Benz(a)anthracene | 0.90 - 30.0 | <2.00 - 80.0 | 0.30 - 30.0 | 50.0 - 360.0 |
| Benzo(a)pyrene | 1.00 - 10.0 | <3.00 - 160.0 | <4.0×10^-3^ - 180.0 | 340.0 |
| Benzo(b)fluoranthene | 1.00 - 60.0 | <3.00 - 260.0 | - | 40.0 - 520.0 |
| Benzo(k)fluoranthene | 1.00 - 20.0 | <3.00 - 90.0 | - | 110.0 - 370.0 |
| Benzo(b+k)fluoranthene | - | - | 1.90 - 610.0 | - |
| Chrysene | 1.00 - 50.0 | <2.0- 200.0 | 1.09 - 290.0 | 100.0 - 620.0 |
| Dibenz(a,h)anthracene | 1.00 - 2.00 | <5.00 - 20.0 | <5.0×10^-3^ - 50.0 | 110.0 |
| Indeno(1,2,3-c,d)pyrene | 1.00 - 30.0 | <5.00 - 160.0 | 2.1×10^-2^ - 290.0 | 310.0 |
| Naphthalene | 2.00 - 160.0 | 2.00 - 40.0 | - | 260.0 - 970.0 |
