# Supplementary material for: Environmental contamination with polycyclic aromatic hydrocarbons and contribution from biomonitoring studies to the surveillance of global health
Source: Environ Sci Pollut Res Int. 2024 Aug 29;31(42):54339–62. doi: 10.1007/s11356-024-34727-3 (PMC11413127; doi:10.1007/s11356-024-34727-3)
Supplement: Supplementary file 7 — Supplementary file7 (DOCX 227 KB) [file 11356_2024_34727_MOESM7_ESM.docx]

**Online Resource 7**

Environmental contamination with polycyclic aromatic hydrocarbons and contribution from biomonitoring studies to the surveillance of global health

Joana Teixeira, Cristina Delerue-Matos, Simone Morais, Marta Oliveira*

REQUIMTE/LAQV, ISEP, Polytechnique of Porto, Rua Dr. António Bernardino de Almeida 431, 4249-015, Porto, Portugal

*Corresponding author: Tel.: +351 22 834 0500

E-mail: *marta.oliveira@graq.isep.ipp.pt*

| 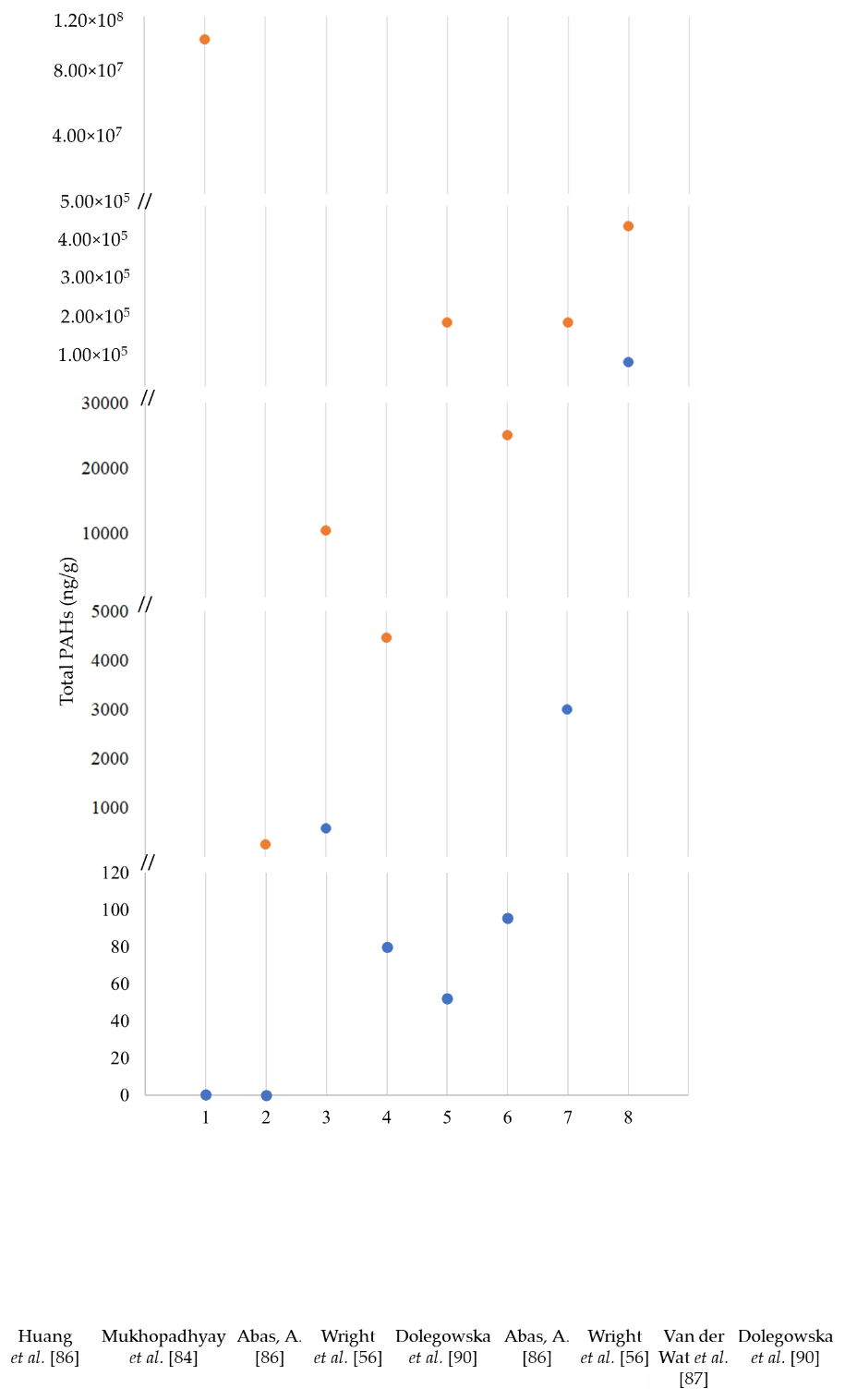 | 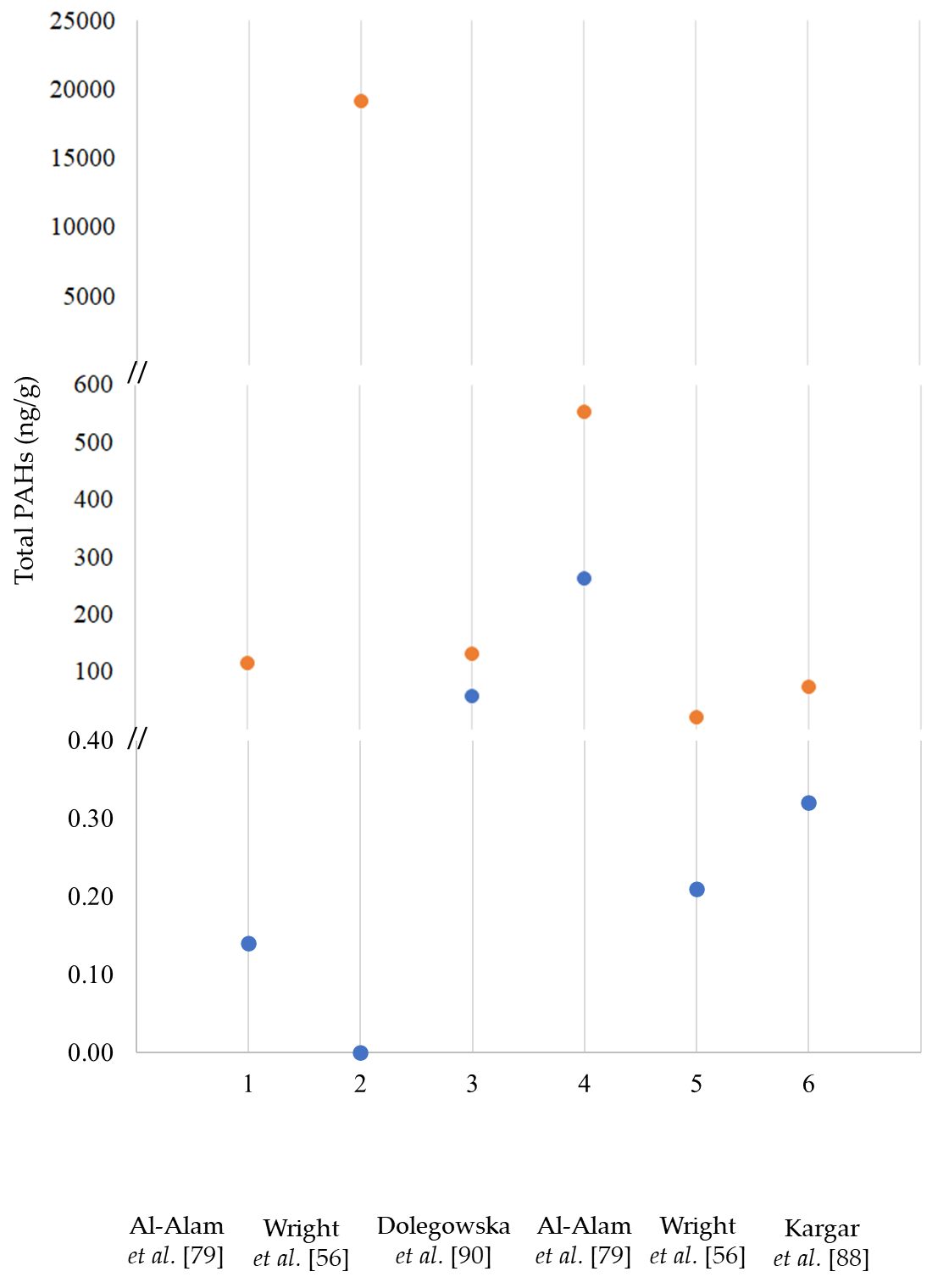 |
| --- | --- |
| a) | b) |

Levels of total PAHs (minimum – maximum, represented as blue and orange dots, respectively) reported in a) mosses, lichens [1 – Mukhopadhyay et al., 2020; 2 and 5 – Abas, 2021; 3 and 6 - Wright et al., 2018; 4 and 8 - Dolegowska et al., 2021; 7 - Van der Wat and Forbes, 2015] b) conifer needles and leaves [1 and 4 – Al-Alam et al., 2019; 2 and 5 – Wright et al., 2018; 3 – Dolegowska et al., 2021; 6 – Kargar et al., 2017].
