# Supplementary material for: Environmental contamination with polycyclic aromatic hydrocarbons and contribution from biomonitoring studies to the surveillance of global health
Source: Environ Sci Pollut Res Int. 2024 Aug 29;31(42):54339–62. doi: 10.1007/s11356-024-34727-3 (PMC11413127; doi:10.1007/s11356-024-34727-3)
Supplement: Supplementary file 8 — Supplementary file8 (DOCX 53 KB) [file 11356_2024_34727_MOESM8_ESM.docx]

**Online Resource 8**

Environmental contamination with polycyclic aromatic hydrocarbons and contribution from biomonitoring studies to the surveillance of global health

Joana Teixeira, Cristina Delerue-Matos, Simone Morais, Marta Oliveira*

REQUIMTE/LAQV, ISEP, Polytechnique of Porto, Rua Dr. António Bernardino de Almeida 431, 4249-015, Porto, Portugal

*Corresponding author: Tel.: +351 22 834 0500

E-mail: *marta.oliveira@graq.isep.ipp.pt*


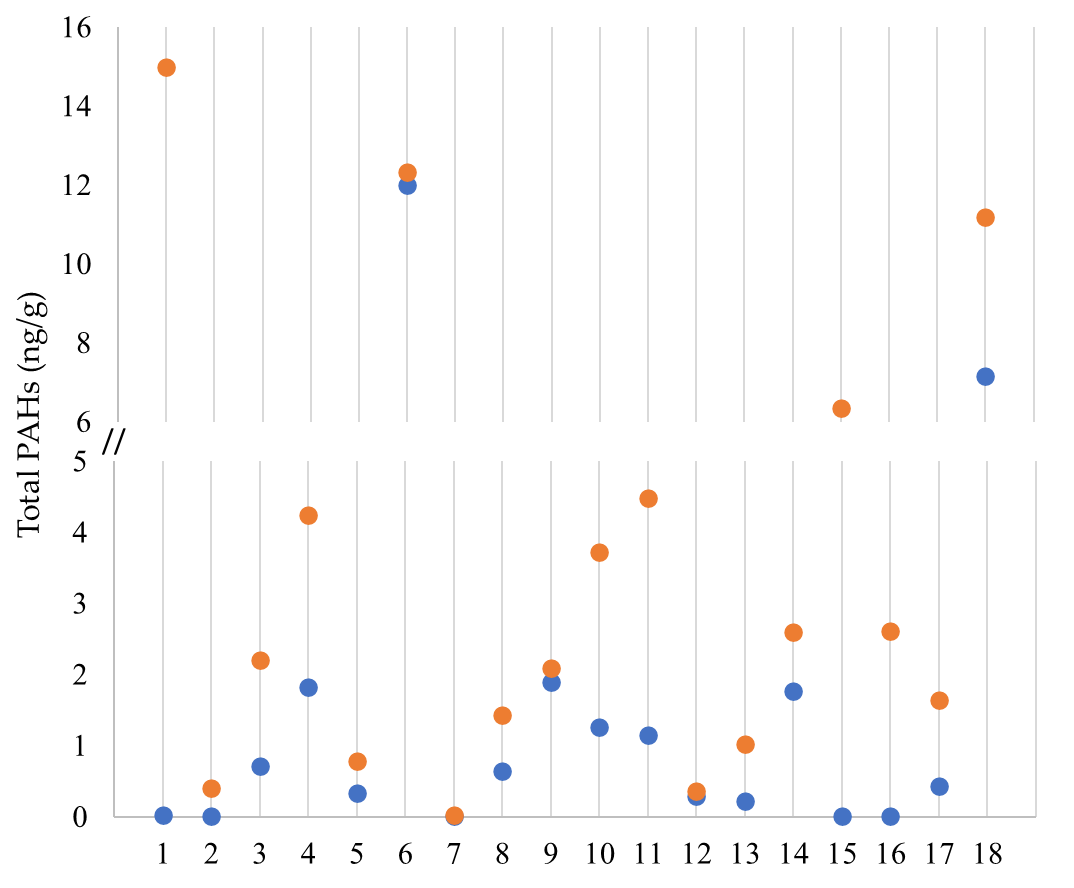


Levels of total PAHs (minimum – maximum, represented as blue and orange dots, respectively) reported in fruits [1 – Apple; 2 – Apricot; 3 – Banana; 4 – Cherry; 5 – Grape; 6 – Grapefruit; 7 – Gooseberry; 8 – Guava; 9 – Lemon; 10 – Mandarin; 11 – Nectarine; 12 – Orange; 13 – Papaya; 14 – Peach; 15 – Pear; 16 – Quince; 17 – Strawberry; 18 – Kiwi] (Paris *et al.*, 2018).
