# Supplementary material for: Environmental contamination with polycyclic aromatic hydrocarbons and contribution from biomonitoring studies to the surveillance of global health
Source: Environ Sci Pollut Res Int. 2024 Aug 29;31(42):54339–62. doi: 10.1007/s11356-024-34727-3 (PMC11413127; doi:10.1007/s11356-024-34727-3)
Supplement: Supplementary file 9 — Supplementary file9 (DOCX 201 KB) [file 11356_2024_34727_MOESM9_ESM.docx]

**Online Resource 9**

Environmental contamination with polycyclic aromatic hydrocarbons and contribution from biomonitoring studies to the surveillance of global health

Joana Teixeira, Cristina Delerue-Matos, Simone Morais, Marta Oliveira*

REQUIMTE/LAQV, ISEP, Polytechnique of Porto, Rua Dr. António Bernardino de Almeida 431, 4249-015, Porto, Portugal

*Corresponding author: Tel.: +351 22 834 0500

E-mail: *marta.oliveira@graq.isep.ipp.pt*


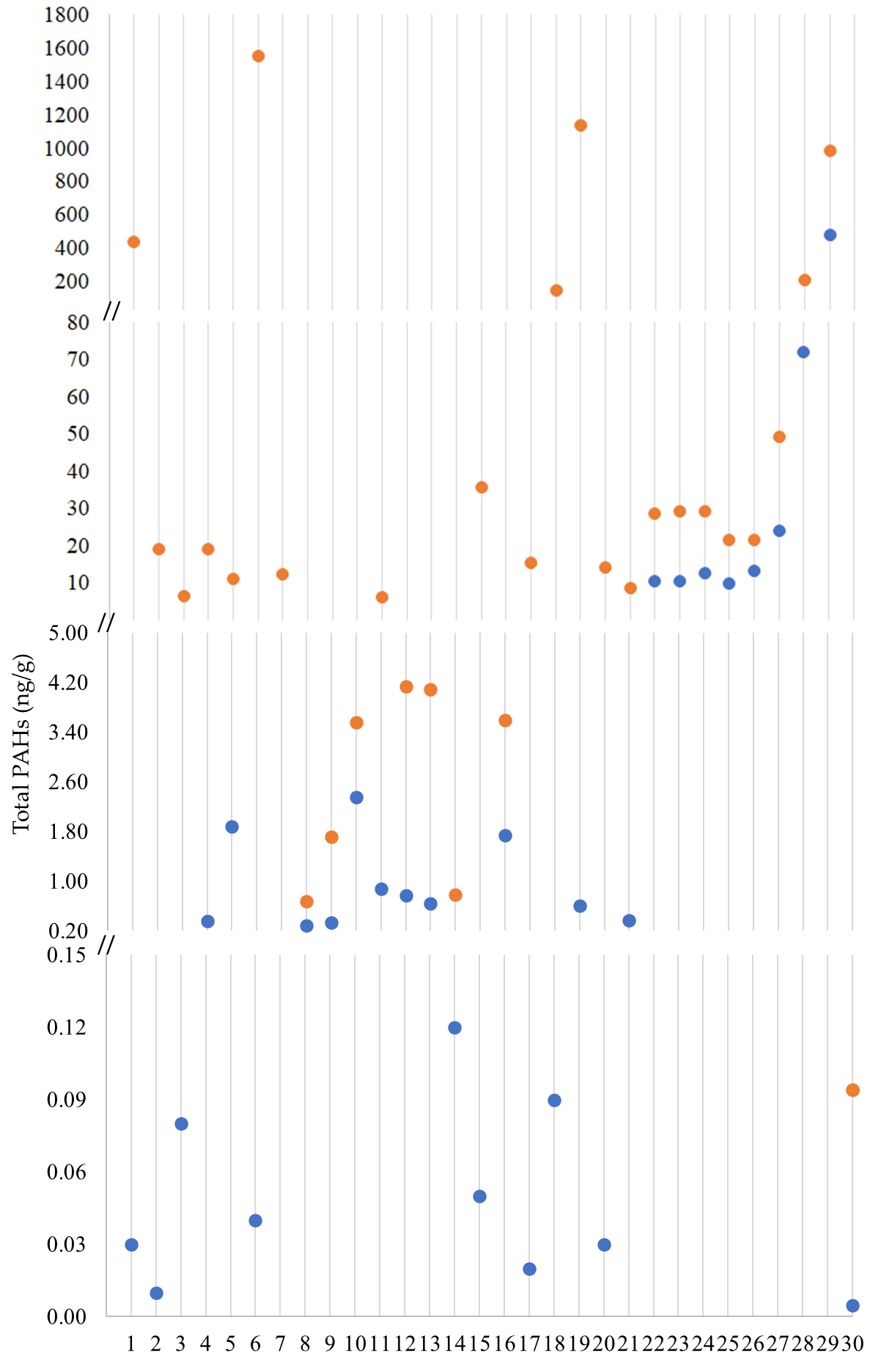


Levels of total PAHs (minimum – maximum, represented as blue and orange dots, respectively) reported in vegetables [1 – Cabbage; 2 – Carrot; 3 – Cauliflower; 4 – Celery; 5 – Colocasia; 6 – Cucumber; 7 – Eggplant; 8 – Endive; 9 – Fenugreek; 10 – Garlic; 11 – Gourd; 12 – Kale; 13 – Kohlrabi; 14 – Leek; 15 – Lettuce; 16 – Onion; 17 – Potato; 18 – Radish; 19 – Spinach; 20 – Tomato; 21 – Turnip (Paris *et al.*, 2018); 22 – Potato; 23 – Cucumber; 24 – Tomato; 25 – Eggplant; 26 – Wax gourd; 27 – Celery; 28 – Root; 29 – Aerial; 30 – Lettuce (Yebra-Pimentel *et al.*, 2015).
