# Supplementary material for: Environmental contamination with polycyclic aromatic hydrocarbons and contribution from biomonitoring studies to the surveillance of global health
Source: Environ Sci Pollut Res Int. 2024 Aug 29;31(42):54339–62. doi: 10.1007/s11356-024-34727-3 (PMC11413127; doi:10.1007/s11356-024-34727-3)
Supplement: Supplementary file 10 — Supplementary file10 (DOCX 161 KB) [file 11356_2024_34727_MOESM10_ESM.docx]

**Online Resource 10**

Environmental contamination with polycyclic aromatic hydrocarbons and contribution from biomonitoring studies to the surveillance of global health

Joana Teixeira, Cristina Delerue-Matos, Simone Morais, Marta Oliveira*

REQUIMTE/LAQV, ISEP, Polytechnique of Porto, Rua Dr. António Bernardino de Almeida 431, 4249-015, Porto, Portugal

*Corresponding author: Tel.: +351 22 834 0500

E-mail: *marta.oliveira@graq.isep.ipp.pt*

**
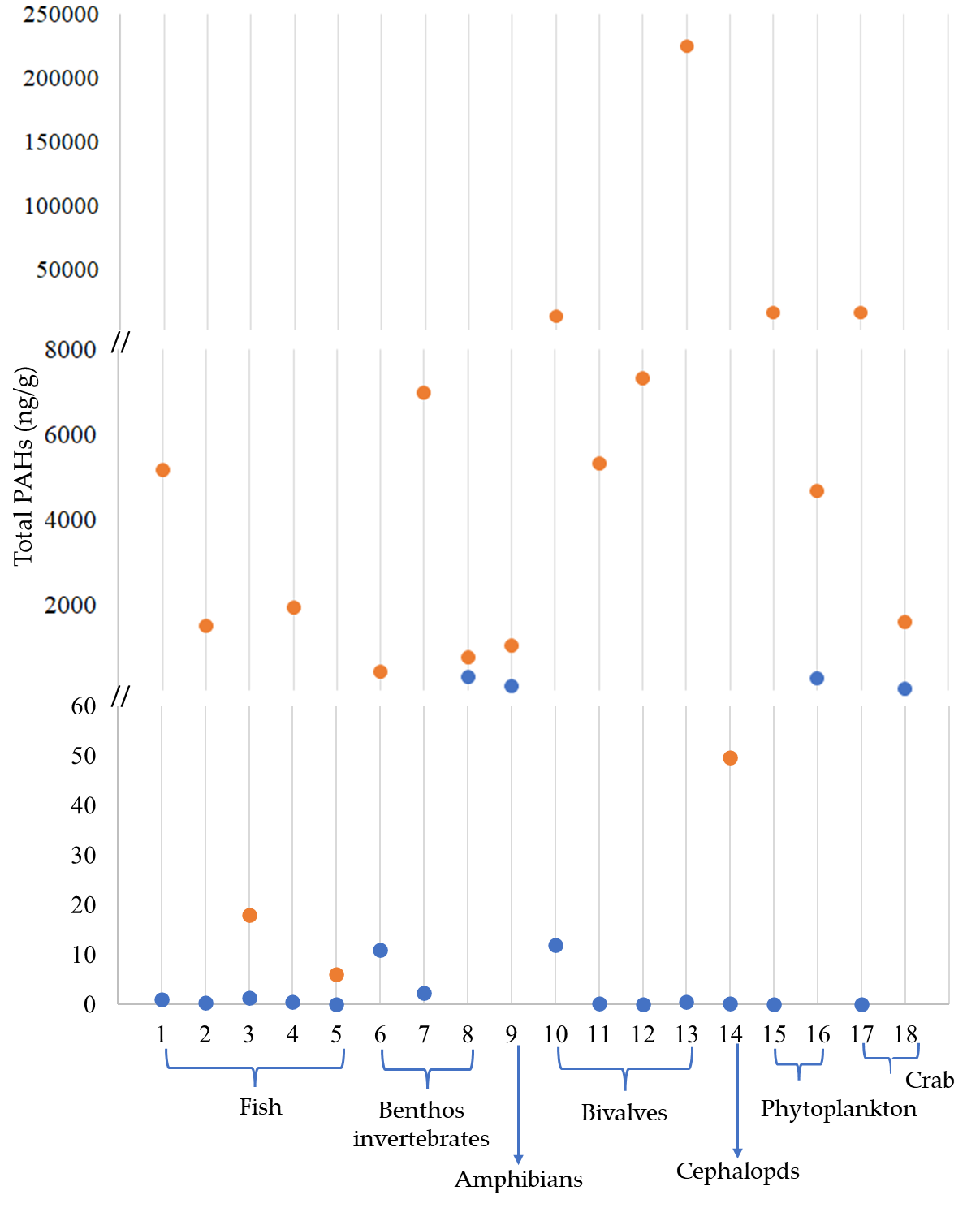
**

Levels of total PAHs (minimum – maximum, represented as blue and orange dots, respectively) reported in fish, benthos invertebrates, amphibians, bivalves, cephalopods, phytoplankton, and crab [1, 6, 9 and 10 – Wallace et al., 2020; 2, 7, 12 and 17 – Honda et al., 2020; 3 – Oliveira et al., 2020; 4, 8, 13, 16 and 18 – Yebra-Pimentel et al., 2015; 5 – Srogi et al., 2007; 11 and 15 – Thuy et al., 2018; 14 – Gomes et al., 2013]
