# Supplementary material for: Environmental contamination with polycyclic aromatic hydrocarbons and contribution from biomonitoring studies to the surveillance of global health
Source: Environ Sci Pollut Res Int. 2024 Aug 29;31(42):54339–62. doi: 10.1007/s11356-024-34727-3 (PMC11413127; doi:10.1007/s11356-024-34727-3)
Supplement: Supplementary file 11 — Supplementary file11 (DOCX 124 KB) [file 11356_2024_34727_MOESM11_ESM.docx]

**Online Resource 11**

Environmental contamination with polycyclic aromatic hydrocarbons and contribution from biomonitoring studies to the surveillance of global health

Joana Teixeira, Cristina Delerue-Matos, Simone Morais, Marta Oliveira*

REQUIMTE/LAQV, ISEP, Polytechnique of Porto, Rua Dr. António Bernardino de Almeida 431, 4249-015, Porto, Portugal

*Corresponding author: Tel.: +351 22 834 0500

E-mail: *marta.oliveira@graq.isep.ipp.pt*

**
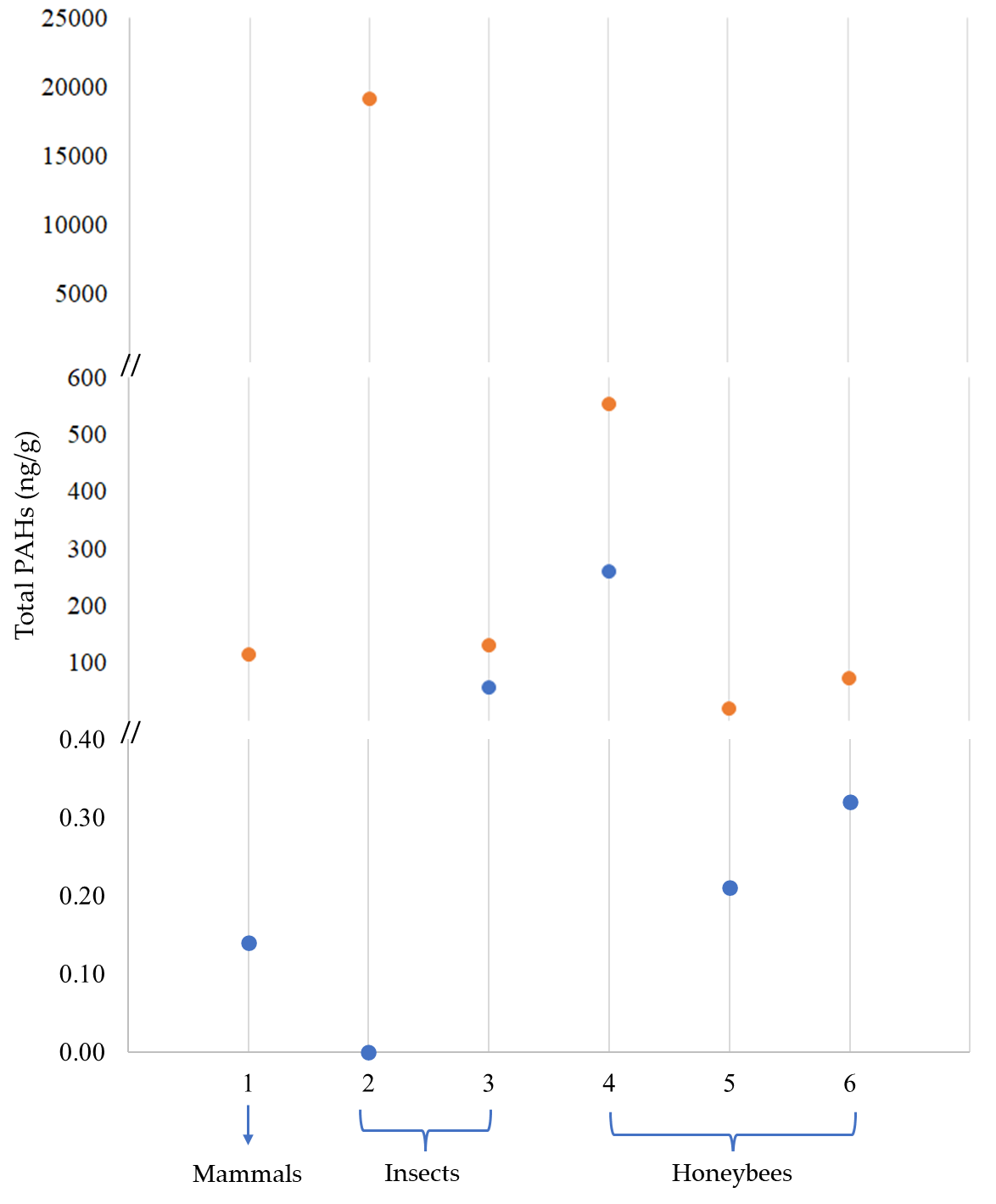
**

Levels of total PAHs (minimum – maximum, represented as blue and orange dots, respectively) reported in terrestrial ecosystems: mammals and insects including honeybees [1, 2 – Wallace et al., 2020; 3 – Honda et al., 2020; 4 – Kargar et al., 2017; 5 – Cochard et al., 2021; 6 – Lambert et al., 2012]
